# Wound Healing of Cutaneous Sulfur Mustard Injuries: Strategies for the Development of Improved Therapies

**Published:** 2005-01-05

**Authors:** John S. Graham, Robert P. Chilcott, Paul Rice, Stephen M. Milner, Charles G. Hurst, Beverly I. Maliner

**Affiliations:** a Comparative Pathology Branch, Comparative Medicine Division and Chemical Casualty Care Division, U.S. Army Medical Research Institute of Chemical Defense, Aberdeen Proving Ground, MD; b Department of Biomedical Sciences, Defence Science and Technology Laboratory, Porton Down, Salisbury, Wiltshire, UK; c The Plastic Surgery Institute, Southern Illinois University School of Medicine, Springfield

## Abstract

Sulfur mustard is an alkylating chemical warfare agent that primarily affects the eyes, skin, and airways. Sulfur mustard injuries can take several months to heal, necessitate lengthy hospitalizations, and result in significant cosmetic and/or functional deficits. Historically, blister aspiration and/or deroofing (epidermal removal), physical debridement, irrigation, topical antibiotics, and sterile dressings have been the main courses of action in the medical management of cutaneous sulfur mustard injuries. Current treatment strategy consists of symptomatic management and is designed to relieve symptoms, prevent infections, and promote healing. There are currently no standardized or optimized methods of casualty management that prevent or minimize deficits and provide for speedy wound healing. Several laboratories are actively searching for improved therapies for cutaneous vesicant injury, with the aim of returning damaged skin to optimal appearance and normal function in the shortest time. Improved treatment will result in a better cosmetic and functional outcome for the patient, and will enable the casualty to return to normal activities sooner. This editorial gives brief overviews of sulfur mustard use, its toxicity, concepts for medical countermeasures, current treatments, and strategies for the development of improved therapies.

## HISTORICAL PERSPECTIVE OF SULFUR MUSTARD USE

Sulfur mustard [bis(2-chloroethyl)sulfide; or HD] is a potent vesicating (blistering) chemical warfare agent that was first used in the First World War by Germany against French troops at Ypres, Belgium (1917). Since then, there has been evidence or allegations of HD use in 11 conflicts, including by Italy against Ethiopia in 1936, by Japan against China in 1937, by Poland against Germany in 1939, by Egypt against Yemen from 1963 to 1967, and by Iraq against Iran in the 1980s.^1^ In addition, its use was threatened in the early 1990s during the Persian Gulf War. There are no peacetime industrial uses for HD. Aging HD stocks remain hazardous to civilians. These munitions and stockpiles were discarded on land and into the ocean during and after the Second World War. Periodically they surface during agricultural or fishing activities, causing serious injury. Extensive production and stockpiling during the Second World War, along with the known effects of HD on epithelial tissues, have spurred agreements to ban its production and use. These agreements include the 1993 Chemical Weapons Convention (CWC)^2^ and agent destruction programs. Nonetheless, this chemical warfare agent remains a threat owing to its popularity in some countries that are not signatories to the CWC and to accidental exposures. Dispersed as a vapor, aerosol, or in liquid droplets, HD remains a threat to war fighters and civilians worldwide.

As a class, vesicants include sulfur mustard, the nitrogen mustards (HN_1_, the chemotherapeutic agent HN_2_ or “Mustargen,” and HN_3_), arsenicals such as Lewisite, and halogenated oximes such as phosgene oxime. A plethora of information on vesicants is available in the published literature. This report is a review of some of the literature available on HD.

## SULFUR MUSTARD TOXICITY

### General toxicology

The known molecular mechanisms of action, chemistry, toxicodynamics, and genotoxicity of HD as well as the pathogenesis and histopathology of HD injuries have been widely described.^1^,^3^–^16^ Because its actions partly resemble those of ionizing radiation, HD is sometimes considered a radiomimetic compound. Papirmeister et al^1^ wrote an extensive review of HD research, offered several theories of its cytotoxicity, and summarized what is known of its absorption, distribution, biotransformation, and excretion. Somani et al^3^ have also provided an excellent overview of the toxicodynamics of HD.

Current research is not only investigating various aspects of Papirmeister's theories, but also actively pursuing the development and use of topical skin protectants, decontaminating agents, prophylactic and therapeutic countermeasures, and improved therapies for established lesions. Most of the research to date has focused on mitigating cutaneous injury. Larger knowledge gaps exist for injury to the eyes and the respiratory system, and for systemic effects.

### General clinical manifestations

Sulfur mustard is a bifunctional alkylating agent that causes extensive incapacitating injuries to the skin, respiratory tract, and eyes. It reacts with a large variety of molecules of biologic interest, ranging from compounds of low molecular weight to macromolecules such as DNA, RNA, and proteins.^1^ Similar to other alkylating agents, it can cross-link complementary strands of DNA and thereby inhibit cell division. Although injury occurs very rapidly after tissue contact with HD, clinical signs of injury are delayed from 2 to 24 hours following exposure, with the length of the delay being inversely proportional to exposure level and other factors.^16^ Because of this delay and lack of pain on contact, exposures can go unrecognized, and exposed persons may often fail to take immediate decontamination and protective measures, resulting in extensive exposure and severe injury.

Characteristic lesions are a function of dose and time after exposure.^1^ At low toxic doses, skin lesions are characterized by an initial asymptomatic period of highly variable duration followed by localized pruritic erythema.^1^,^15^ Higher doses initially produce small vesicles within or on the periphery of the erythematous areas that may later merge to form large, pendulous blisters.^15^ Vesication may take several days to complete. Very high doses produce coagulation necrosis, which is often characterized as “doughnut burns” owing to the appearance of a central necrotic region of skin surrounded by a circular periphery of less damaged tissue.^15^ Secondary bacterial infections of this type of wound pose a serious threat. In the eyes, low vapor exposures are characterized by reddening. As the concentration increases, conjunctivitis becomes progressively more severe, with photophobia, blepharospasm, pain, and corneal damage seen at higher doses.^1^,^15^ Liquid or very large vapor exposure to the eyes results in severe corneal damage, with potential for blindness.^15^ Inhalation of HD vapor induces changes in laryngeal and tracheobronchial mucosa, with mild to severe inflammation.^1^,^15^ Depending on the degree of exposure, symptoms can range from a mild irritation of the upper respiratory tract to severe bronchiolar damage leading to epithelial necrosis, hemorrhage, inflammatory exudate, and pseudomembrane formation in the tracheobronchial tree.^1^,^15^ While rare, massive pulmonary exposure can result in hemorrhagic pulmonary edema. In the First World War, respiratory injury from vapor exposures resulted in death due to pneumonia secondary to chemical pneumonitis.^15^ More profound respiratory injury from aerosol exposures, as in the Iran-Iraq war of the late 1980s, have resulted in 2 waves of death.^17^ The first wave occurred within 3 days of the attack from respiratory failure due to extreme injury to respiratory epithelium and alveoli. The second wave of deaths occurred between 1 and 3 weeks postexposure and resulted from secondary bronchopneumonia and sepsis secondary to marrow failure. High doses may produce a systemic toxicity that includes destruction of bone marrow precursor cells. Leukopenia typically begins 3 to 5 days after exposure^15^ and may progress to pancytopenia. Very high doses may induce destruction of lymphoid organs and the mucosa of the small intestine and produce effects in the central nervous system, including apathy, depression, hyperexcitability, abnormal muscular movements, and convulsions.^1^,^15^

Sulfur mustard has been shown to be carcinogenic and mutagenic in animal studies, and epidemiological studies of factory workers involved in HD production who were chronically exposed to low doses of HD have implicated it in human cancers (primarily respiratory carcinomas).^1^,^15^,^16^ Skin cancers have been experimentally generated in rats and mice exposed to HD and can potentially arise in HD-induced scar tissue in humans.^15^,^16^ There is no evidence that HD is a cause for adverse reproductive events, although epidemiological study of heavily affected civilian populations may alter current understanding.

Persistent problems of soldiers and factory workers exposed during the First World War include chronic bronchitis, asthma, laryngitis, recurrent pneumonia, and long-term keratitis.^16^ Recent clinical reports of follow-up examinations of survivors of the 1987 Iraqi attack on Sardasht, including those who were children at the time, indicate chronic dermatological, ophthalmic, and respiratory problems.^17^,^18^ Pulmonary dysfunction was the most common problem reported. It was noted that chronic effects tended to be significantly more pronounced in adults, possibly because mechanisms of cellular repair are more dynamic in children.^18^

### Hypotheses of cytotoxicity

There are several hypotheses of HD cytotoxicity: (1) the poly(ADP-ribose) polymerase hypothesis, (2) the thiol-Ca^2+^ hypothesis, and (3) the lipid peroxidation hypothesis.^1^ None of these hypotheses has been fully accepted, and the mechanisms proposed by these theories may be active at the same time. At present, the exact mechanism of action of HD is not known.

The first hypothesis, originally proposed by Papirmeister,^1^ has been coined the poly(ADP-ribose) polymerase (PADPRP) hypothesis. Sulfur mustard injury is initiated by a rapid alkylation of DNA. Alkylated DNA purines undergo spontaneous enzymatic depurination, resulting in numerous apurinic sites that are subsequently cleaved via apurinic endonucleases to form DNA breaks. Accumulation of these breaks activates the chromosomal enzyme PADPRP, which uses NAD^+^ as a substrate to ADP-ribosylate several nuclear proteins, resulting in a depletion of cellular NAD^+^. NAD^+^ depletion appears to be the result of an accelerated rate of use, as opposed to interference with its synthesis. This depletion results in the inhibition of glycolysis and subsequent stimulation of the NADP^+^-dependent hexose monophosphate shunt (HMS) owing to accumulation of glucose-6-phosphate. Activation of the HMS results in induction and secretion of proteases, which in turn lead to the typical pathology changes noted above. Results from in vivo studies using a human skin graft model and in vitro work using human lymphocytes, human keratinocytes, and human skin organ cultures suggest that NAD^+^ levels that decrease below a critical value become irreversible and injury-producing. Nuclear pathology appears to precede cytoplasmic damage. This nuclear damage is characterized by loss of euchromatin and condensation and margination of heterochromatin.^1^ Severe damage to the nuclear envelope, such as blebbing and breakage, has been demonstrated by electron microscopy, along with the formation of perinuclear vacuoles. The extent of nuclear damage has been shown to be dose- and time-related.^1^

Evidence that may contradict the PADPRP hypothesis includes the facts that (1) NAD^+^ depletion does not occur in rat keratinocytes until the concentration of HD is sufficient to inhibit DNA repair^19^; (2) DNA repair in human keratinocytes may be accomplished within 90 minutes of exposure,^20^ whereas significant NAD^+^ depletion is not observed until 1 to 3 hours postexposure; and (3) elevation or maintenance of NAD^+^ in human keratinocytes does not confer protection.^21^

The second hypothesis of HD cytotoxicity, the thiol-Ca^2+^ theory, is based on a mechanism originally proposed by Orrenius and Nicotera in their studies of rat hepatocytes.^22^ The initiating event here is a reduction in cellular protein thiol levels, leading to toxic increases in free cytosolic Ca^2+^ levels. The thiol levels are depleted as a result of direct reactions of glutathione (GSH) and protein sulfhydryls with oxidants or other electrophilic xenobiotics. Glutathione is an intracellular scavenger of HD, which may be the cause of GSH depletion. Depletion of GSH occurs following reaction with electrophiles, thus exposing protein sulfhydryls to damage by the xenobiotic or by endogenously produced toxic oxygen species that arise as by-products of oxidative metabolism. Alternatively, HD may also react directly with protein thiols to cause the inactivation of enzymes. One group of enzymes affected by modification of its sulfhydryl groups is Ca^2+^ translocases, resulting in alteration of intracellular Ca^2+^ homeostasis. This leads to an increase in cytosolic free Ca^2+^, which in turn leads to activation of Ca^2+^-dependent catabolic processes, including stimulation of proteases, endonucleases, and phospholipases. Protease activity leads to protein degradation and ultimate perturbation of the cytoskeleton. Changes in the cytoskeleton may also arise from direct alkylation by HD on microfilamentous proteins, although this does not appear to play a critical role. Endonuclease activity leads to DNA breaks, leading to chromatin condensation and energy loss in the cell. Activation of phospholipases leads to phospholipid hydrolysis, with subsequent alterations in membrane fluidity and loss of membrane protein function and integrity. The cytoskeletal, nuclear, and membrane changes all lead to cell death.^1^ It is not known whether vesicating doses of HD deplete GSH sufficiently to produce these cytotoxic effects. Slow GSH depletion may be less toxic because it allows time for cell adaptation. The lengthy latent period prior to clinical manifestation of HD lesions is inconsistent with the more rapid injury expected from an alkylation-induced GSH depletion, suggesting that damage to a cellular target other than GSH is responsible for initiating the cytotoxicity and that any involvement of the thiol-Ca^2+^ cytotoxic pathway results from this initial damage.

The third hypothesis involves lipid peroxidation distinct from the thiol-Ca^2+^ hypothesis, where the principal toxic consequence of GSH depletion is the formation of toxic lipid peroxides. Cell death is thus proposed to be due to an accumulation of endogenous oxidants (eg, H_2_O_2_ accumulation resulting in hydroxyl and perferryl ion formation), leading to lipid peroxidation and irreversible membrane damage.^1^

More recent theories to account for the mechanism of HD toxicity include a melanocyte free-radical hypothesis^23^ and one in which HD requires metabolic activation.^24^ Owing to the multiple molecular targets of HD, multiple pathways to cell death may be initiated. These multiple pathways may have some common steps; hence, all of the above hypotheses may play a role in the cytotoxicity of sulfur mustard. Papirmeister^25^ also explained how HD could induce apoptosis (programmed cell death) and postulated how it might contribute to or modify the pathogenesis of HD injury. Kan et al^26^ postulated that HD-induced cell death involves early apoptosis (6–12 hours postexposure) and late necrosis (24 hours), which temporally overlap to produce a single cell-death pathway along an apoptotic-necrotic continuum. Several lines of research are underway to further elucidate the role of apoptosis in HD toxicity.

### Pathogenesis of blisters

The cytotoxic effects of HD on skin have been widely described for a number of species.^1^,^4^,^6^,^10^,^27^–^35^ The primary cutaneous cell population targeted by HD is the basal cell of the epidermis. Blisters (humans) and microblisters (animal models) occur on separation of the epidermis from the dermis at the dermal-epidermal junction. This separation is dependent on the loss of integrity of basal cells and anchoring filaments.^4^ In animal model studies, the development of an apparent initial nuclear pathologic condition of basal cells of the stratum germinativum was followed by progressive cytoplasmic changes, leading to the eventual death of affected basal cells. Petrali et al^4^ described degenerative subcellular effects of HD on the skin of hairless guinea pigs 24 hours after exposure. Degeneration was also observed in human lymphocytes and keratinocytes in vitro by Petrali et al,^4^ in a variety of animal models by Papirmeister et al,^1^,^34^ and in rabbits and guinea pigs by Vogt et al.^6^ While degenerative changes are noted in the upper layers of the epidermis as well as in epidermal cells of hair follicles, changes are most prevalent among basal cells, with the earliest and most severe degeneration seen in cells located above the dermal papillae.^1^ Microblisters are observed to arise from focal areas of epidermal-dermal separation in areas of widespread basal cell pyknosis, 24 to 48 hours after HD exposure, as seen by light microscopy. Progressive changes reported in basal epithelial cells include formation of perinuclear or paranuclear vacuoles, a decrease in nuclear staining intensity, cytoplasmic swelling, relocation of chromatin to the periphery of the nucleus, loss of chromatin, and pyknosis.^33^ These changes are followed or accompanied by necrosis, vacuolization, or hydropic degeneration of the cytoplasm.^33^ While the extent of nuclear damage is dose- and time-related, it does not take place simultaneously in all basal cells examined, probably reflecting differing repair efficiencies of cells in various phases of the cell cycle.^1^ Nuclear pyknosis has also been described following HD exposure in human skin equivalent.^36^,^37^ It was also described in isolated perfused porcine skin flaps exposed to the HD simulant 2-chloroethyl methyl sulfide, a monofunctional analog of HD.^38^ The time of onset of nuclear or cytoplasmic changes is species-specific. In general, pyknotic nuclei begin to appear in the basal cell layer 3 to 6 hours after HD exposure. By 12 hours, focal areas of pyknosis are seen and become widespread by 24 hours, with the nuclei of many suprabasal keratinocytes becoming involved.

The pathogenesis of microblisters is not fully understood. Petrali et al^5^,^39^ found indications that proteins of extracellular matrices of the basement membrane zone (BMZ) are affected during the development of HD-induced skin pathology in hairless guinea pigs and postulated that they may contribute to the formation of microblisters. Immunohistochemical staining for bullous pemphigoid antigen, a noncollagenous protein shared between basal cell hemidesmosomes and the lamina lucida, revealed a diminishing of bullous pemphigoid antigen reactivity at early times and subsequent loss of antigenicity at later time periods after an 8-minute HD vapor exposure (tissues were harvested at selected postexposure time periods up to 24 hours). Laminin, the major glycoprotein of the lamina lucida, showed scanty immunolocalization at the later time periods, conforming to the structurally altered lamina lucida at microblister lesion sites. The reactivity of Type IV collagen, a ubiquitous protein assigned to the lamina densa of basement membranes, was unaltered to specific antisera throughout prevesication and vesication time periods. The influence of these altered macromolecules on repair mechanisms following HD toxicity is not known. Other proteins of the BMZ yet to be investigated or discovered likely play a role in the pathogenesis of HD-induced microblisters.

### Chemical properties

A summary of the chemical, physical, environmental, and biological properties of HD and other vesicating agents (Lewisite, phosgene oxime) can be found in *The Textbook of Military Medicine*.^15^ Sulfur mustard is a pale yellow to dark brown oily liquid, with an odor of garlic or mustard. It has low solubility in water and is highly lipophilic, so it readily partitions into the skin. Rates of 150 μg cm^−2^ min^−1^ through human skin in vivo^40^ and 157 μg cm^−2^ min^−1^ through heat-separated human skin in vitro^41^ have been reported. Penetration is enhanced by moisture and heat, and in thin skin. The LD_50_ of liquid HD is about 100 mg kg^−1^. This is enough fluid (5–6 mL) to cover about 25% total body surface area (TBSA) in an adult.^15^ A 10-μg droplet is enough to cause vesication. The threshold for vapor/aerosol to induce damage is 200 to 2,000 mg min m^−3^ (concentration time, C*t*), dependent on the anatomical location, environmental conditions (temperature and humidity), sweating, and other factors.^15^ Vapor injury generally induces superficial or partial-thickness dermal injury. Liquid HD can produce full-thickness injury. Because of its high freezing point (14°C), HD is very persistent in cold and temperate climates. Its persistence is lower in warmer climates, where the agent vaporizes more easily.

### Clinical dermal effects

Clinically, erythema is the first symptom noted, typically with a delayed onset of 4 to 8 hours. Pruritis, burning, or stinging may accompany the erythema.^15^ Slight edema may also be present. If the exposure was small, the erythema will not progress to vesication. If the lesion progresses, very small vesicles will develop within or on the periphery of the erythematous areas beginning at about 2 to 18 hours postexposure.^15^ These vesicles can later coalesce to form large blisters/bullae. Vesication may take several days to complete. Additional (new) blisters may arise a week later. Extremely high doses may induce a central zone of coagulation necrosis, with blisters forming along the periphery. Sulfur mustard blisters are subepidermal. Smaller ones are quite durable but large bullae are vulnerable to friction. The blister fluid is initially thin and clear, or slightly straw-colored, and later turns yellowish and tends to coagulate. It does not contain unreacted agent and is not a vesicant itself, unlike the fluid in Lewisite-induced blisters.^15^ High environmental temperatures, hydrated skin, thin or delicate skin, or skin occluded by clothing will generally exhibit more severe lesions and shorter times to onset of symptoms than cooler, less hydrated, thicker, or unoccluded skin under similar levels of exposure. The face, neck, antecubital fossae, axillae, perineum, and external genitalia tend to be very sensitive areas. Differences in individual sensitivity to HD have been noted. Skin color has not been shown to affect HD penetration rates.^42^ Transient hyperpigmentation was reported in casualties of the 1980s Iran-Iraq War, primarily due to accumulation of melanin derived from dead melanocytes at the base of an epidermis about to desquamate, and from opacification and darkening of nonviable epidermal cells.^15^,^43^,^44^ Following desquamation, the skin may appear hypopigmented. It is our opinion that long-term hyperpigmentation results from stimulation of melanocytes and hypopigmentation from melanocyte destruction. These pigmentary changes usually reverse in 6 to 12 months, but can be permanent.

Healing time of these dermal injuries depends on severity, with erythema alone taking several days to subside and severe lesions taking weeks to months depending on the anatomical location, depth of injury, and size. Lesions can be painful and secondary bacterial infection is a common problem (only in humans). While full reepithelialization has sometimes been considered as an endpoint for healing, barrier function must return for the lesion to be considered completely healed. Preliminary data from our laboratory indicate that barrier function, as indicated by transepidermal water loss (TEWL) measurements, is severely disrupted in ventral abdominal pig skin by 3 to 7 days after 30 to 120 minutes of exposure to liquid HD. While full reepithelialization of these deep dermal/full-thickness injuries appears grossly to occur 35 to 56 days after exposure, TEWL values do not return to baseline until 63 to 70 days postexposure (J. S. Graham et al, unpublished data, 1998). The delay in barrier disruption following agent exposure can be explained by the in situ existence of an intact stratum corneum for the first few days following exposure. The stratum corneum remains intact for 2 to 3 days, after which barrier function becomes compromised (ie, TEWL rates greatly increase) owing to loss of sloughing epidermis or deroofing of a blister. Following wound closure (eg, complete coverage of the damaged area by migrating keratinocytes), additional time is needed to form a fully stratified epidermis with a well-formed stratum corneum. Poor barrier function is generally attributed to an imbalance in water content, lack of proper organization, and/or adhesion of the corneocytes that make up the stratum corneum.

### Long-term cutaneous effects

Residual health effects of significant HD exposure are usually respiratory, ocular, or cutaneous. The permanent consequences of cutaneous injury can include hypopigmentation, hyperpigmentation, fragile skin that is easily damaged by trauma, and hypertrophic scarring. Skin hypersensitivity and chronic ulceration problems have also been reported.^16^ Hypertrophic scarring is a result of uncontrolled fibroblastic activity and overgrowth of connective tissue during wound repair.^16^ Excessive wound contraction has been noted in a weanling pig model following deep dermal/full-thickness HD injury^45^ and observed in human casualties.^44^ Excessive scarring and/or wound contraction over joints is disfiguring and impedes dexterity and locomotion. If initial treatment is not sufficiently aggressive and excessive wound contraction and scar tissue formation occur, surgical release may be indicated. As it is generally accepted that skin cancers can arise in scar tissue, HD-induced scars can be considered to have a carcinogenic potential. It has previously been noted that cutaneous cancers resulting from acute HD exposures usually localize in scars.^16^ While cutaneous malignancies have been reported, they appear to be an uncommon consequence of HD exposure if indeed they were due to HD exposure alone.^46^ Theories on the mechanism for malignant expression abound.

## CONCEPTS FOR MEDICAL COUNTERMEASURES TO SULFUR MUSTARD

Concepts for medical countermeasures to vesicant agents have been developed and active research programs are underway in a number of North Atlantic Treaty Organization (NATO) countries. Institutes actively involved in this research area include the US Army Medical Research Institute of Chemical Defense, located at Aberdeen Proving Ground, Md, the Biomedical Sciences Department of the Defence Science and Technology Laboratory, Porton Down, Salisbury, Wiltshire, UK, and the Defence Research and Development Canada—Suffield Medicine Hat, Alberta. Developed research concepts include elimination of body contact, improved decontamination, pharmacological intervention, and chemical casualty management.

### Elimination of contact

The initial step in protecting a person from the deleterious effects of HD is to eliminate contact with the agent. Respirators/protective masks can be donned to eliminate respiratory and ocular exposures,^47^ specialized protective clothing (including suit, gloves, and foot protection) can be donned to prevent the agent from reaching the skin,^47^ and topical skin protectants^48^–^62^ can be used to protect areas of the skin vulnerable to the agent (eg, wrist, ankle, waist, and neck junctions in protective clothing).

### Decontamination

Skin injury from HD can be completely avoided through rapid physical removal, ideally within minutes. Removal in less than 2 minutes completely prevents damage, but even later removal lessens injury.^15^ Physical removal without agent neutralization (eg, wiping with cloth or gauze, scraping with tongue depressor) can remove bulk agent from the skin and help reduce the severity of any resultant lesion. Copious quantities of water with soap appear to be quite effective in physically removing liquid HD from the skin surface.^63^ Decontamination can also be performed by physical adsorption with or without chemical inactivation. There are a number of decontaminating agents available to remove the agent that are based on reactive powders (eg, Ambergard XE-555 Resin [Rohm and Haas Company, Philadelphia, Pa]^63^), neutralizing solutions (eg, 0.5% hypochlorite^63^), reactive skin lotions (eg, Reactive Skin Decontamination Lotion [RSDL, O'Dell Engineering Ltd/E-Z-EM, Inc, Lake Success, NY]^64^), or absorbent powders (eg, fuller's earth). Equally effective, however, are commonly available household products such as baking flour followed by wet wipes.^65^ Solvents such as kerosene and surgical spirit may also be effective for removing HD from skin.^66^

Wound healing studies using weanling swine have demonstrated that following exposure to undiluted liquid HD for 120 minutes, there is a significant period of off-gassing of unbound agent, as measured by a MINICAMS air monitor (OI Analytical, College Station, Tex; J. S. Graham et al, unpublished data, 2001). Quantification and localization of the HD depot responsible for this lengthy off-gassing in this animal model has not been performed. The existence of a dermal reservoir of HD in humans was first suggested in World War 1 by Smith et al,^66^ who demonstrated that HD injuries could be prevented by washing contaminated skin with an appropriate solvent up to 45 minutes postexposure.

Furthermore, Smith et al demonstrated that the skin reservoir of HD could be transferred to a second individual, even after the exposed surface had been decontaminated. However, studies conducted during the Second World War reported the opposite effect in that HD was rapidly “fixed” by skin constituents such as proteins.^40^ Contemporary in vitro studies have confirmed the original finding of Smith et al that a substantial reservoir of HD is formed in human skin that can account for up to 35% of the applied dose after 24 hours.^41^ This reservoir has been localized to the stratum corneum and upper epidermis. This substantiates work conducted by Cullumbine^67^ which demonstrated that the process of vesication could be blocked by the timely application of “peeling” (keratinlytic) agents up to 14 hours postexposure. The existence of an HD depot in human skin for a period of time following exposure has implications for the safety of medical emergency personnel treating HD casualties; for example, use of protective apparel may be warranted. Prior to medical treatment or casualty transport in enclosed vehicles, thoroughness of cleansing should be ensured through multiple washings and/or use of a detector. The existence of an agent depot could also influence the design of decontaminating agents. Any decontaminating agent that is capable of pulling the targeted agent out of the skin as well as neutralizing it on the surface of the skin has the potential to decrease HD-induced pathology, even beyond the 2-min efficacy window^15^ of conventional decontamination procedures.

Understanding the kinetics of HD skin absorption, and the amount and persistence of unbound HD in putative agent reservoirs, will aid in choosing the most appropriate decontaminating agent and in determining its application doctrine and its window of effective use. The best agent will be the one that not only decontaminates HD sitting on the surface of the skin, but also is capable of fully penetrating the stratum corneum and neutralizing any unbound agent reservoir located there, unlike previous reactive therapies.^68^ Research is underway to develop an in vitro model for efficacy testing of advanced decontaminating agents capable of pulling HD out of the skin reservoir and neutralizing HD on the surface of the skin, to identify the most appropriate animal model for extrapolation of animal data to humans, and to conduct in vivo efficacy tests of candidate decontamination systems.

### Pharmacological intervention

Because HD is not painful on contact, the exposed person may not be aware of the exposure until symptoms begin to appear after the latent period. Pharmacological approaches are being studied for their efficacy in minimizing or preventing that damage.^69^–^71^ The most successful strategies to date have included the use of anti-inflammatories, protease inhibitors, intracellular scavengers, cell cycle inhibitors, PADPRP inhibitors, and calcium modulators, (Dr William J. Smith, PhD, US Army Medical Research Institute of Chemical Defense, Aberdeen Proving Ground, Md, oral communication, 2004). It is interesting to note that pretreatments (eg, antioxidants) that reside in the extracellular matrix are generally more effective than those designed for intracellular protection against HD (Defence Science and Technology Laboratory, Porton Down, unpublished data, 2004). This may imply that the cytotoxic effects of HD are mediated via interaction with cell surface components rather than intracellular targets. Thus, further investigations of the interaction of cell-surface molecules with HD may provide a new insight into the mechanisms of HD toxicity.

### Chemical casualty management

When HD comes in contact with the skin, decontamination is not performed in a timely fashion, and pharmacological intervention is absent or not adequately effective, a chemical casualty will be produced that requires medical attention. Casualty management now comes into play, discussed in depth in the next section. Educational material and training courses are available through the Chemical Casualty Care Division of the US Army Medical Research Institute of Chemical Defense, Aberdeen Proving Ground, Md (on the Web at https://ccc.apgea.army.mil/default.htm, by phone at 410-436-2230/3393, or by e-mail at ccc@apg.amedd.army.mil).

## CURRENT TREATMENTS FOR CUTANEOUS SULFUR MUSTARD INJURIES

There are currently no standardized or optimized methods of casualty management and no drugs available to prevent the effects of HD on skin and mucous membranes. Historically, blister aspiration and/or deroofing (epidermal removal), physical debridement, irrigation, topical antibiotics, and sterile dressings have been the main courses of action in the medical management of cutaneous HD injuries.^12^,^14^,^15^,^72^,^73^ Current treatment strategy consists of symptomatic management and is designed to relieve symptoms, prevent infections, and promote healing. The general recommendations listed below are fully described in the *Textbook of Military Medicine*,^15^ the *Field Management of Chemical Casualties Handbook*,^74^ the *Medical Management of Chemical Casualties Handbook*,^75^ and the *NATO Handbook on the Medical Aspects of NBC Defensive Operations*.^76^

The decision to evacuate and hospitalize an HD casualty is based on the extent and severity of the skin lesions, in consideration with other injuries that may be present (eg, respiratory, ocular). For patients experiencing only cutaneous HD injuries, erythema covering more than 5% TBSA in noncritical areas requires hospitalization. Erythema covering less than 5% TBSA may require hospitalization, depending on the site of the injury (eg, face, inguinal area) and level of impairment (eg, limitation of limb movement due to pain, edema). Total body surface area can be determined using Wallace's Rule of Nines and the Lund and Browder chart for estimating burn severity.^77^,^78^ Multiple or large areas of vesication will also require hospitalization. Since blister formation may initially be slight, the patient should be watched for a progression in the size and number of blisters. Topical antibacterial creams such as silver sulfadiazine or 10% mafenide acetate can be prescribed to patients not requiring close medical monitoring, with instructions to apply a thin layer to the affected area 4 times a day. Following application of the cream, the area should be covered with a petrolatum gauze bandage.

Not all burn injuries require specialized care in a burn center. The American Burn Association has well-defined criteria for patient transfer to such a center,^78^ which should serve as additional guidance in deciding where to hospitalize an HD casualty.

Before commencement of any treatment, clothing should be carefully removed and treated as potentially contaminated, and the patient thoroughly decontaminated. For a general overview of decontamination procedures, the reader is directed to Hurst's chapter on decontamination in the *Textbook of Military Medicine*.^63^

Direct comparisons in the literature between HD and thermal burns are scarce. Papirmeister noted that disintegration of the basal cell layer caused by thermal burns has been shown to produce an intraepidermal blister that contains fragments of the basal cell layer attached to the basal lamina, unlike the almost totally denuded basement membrane in HD lesions.^1^ An argument against the adage “a burn is a burn” is that HD initially targets a specific cell type (epithelial basal cells), unlike a thermal burn that begins damage at the stratum corneum and then works its way downward. Since stratum corneum is the structure largely responsible for barrier function, water loss rates are very high immediately after a thermal burn (140–180 g m^−2^ h^−1^ in humans).^79^ The stratum corneum remains intact for 2 to 3 days after a cutaneous HD injury, after which barrier function becomes compromised by loss of sloughing epidermis or deroofing of the blister. The systemic fluid derangement seen in cutaneous HD injury is less than that seen with thermal burns. Fluids and electrolytes should be closely monitored, since fluids may be lost to edematous areas, with resultant dehydration. Medical personnel are cautioned not to overhydrate the patient; hypervolemia and pulmonary edema can be iatrogenically induced in HD casualties.^15^,^44^ Fluid requirements in Iranian casualties during the Iran-Iraq war appeared to have been relatively independent of TBSA.^44^ The recommended infusion rates and formulas used to calculate total volume requirements for thermal burn patients, based on body weight and TBSA,^80^ should not be routinely applied in HD casualty management. The exact fluid replacement requirements for cutaneous HD injuries should be based on patient status and considered on a case-by-case basis. The fluids used in replacement fluid therapy for non-HD burns, which would likely be appropriate for use in HD injuries if fluid replacement is required, are described by Settle,^80^ Brisebois,^81^ and Thomas et al.^82^

Sulfur mustard casualties should be kept comfortable and their lesions regularly cleansed to prevent infections. Limbs may need to be immobilized, as movement of joints can aggravate existing lesions. Blisters arising on the trunk require protective dressings to avoid or minimize damage as a result of friction with clothing or bedding. As burning and itching sensations are typically present after the appearance of erythema is noted, topical antipruritics are applied (eg, calamine lotion, 0.25% camphor, menthol, corticosteroidal preparations, and silver sulfadiazine cream). Systemic analgesics and antipruritics may be indicated, depending on the discomfort level of the patient.

Infection is a significant factor in causing delayed healing of cutaneous HD injuries. These injuries are covered by necrotic debris, which is a nidus for infection. There is no consensus, however, on whether intact blisters should be deroofed. Blister fluid from intact blisters provides a sterile wound covering, but the blisters are fragile and easily ruptured. Once blisters have broken, ragged roofs should be removed and sterile dressings put in place as soon as possible. Wounds should be inspected periodically for signs of infection.

It is generally recommended in military medical manuals to deroof blisters that are greater than 1 cm in diameter, irrigating the underlying area 2 to 4 times per day with saline, sterile water, clean soapy water, or Dakin's solution. Following cleansing, the area should be liberally covered with a topical antibiotic cream (eg, silver sulfadiazine; mafenide acetate; bacitracin; and triple combination preparations of neomycin sulfate, polymyxin B sulfate, and bacitracin zinc [Neosporin, Pfizer Inc, New York, NY]). A sterile dressing should then be put in place. Blisters that have already broken should have their ragged edges removed and the area irrigated, treated with antibiotic, and dressed with a sterile dressing. Blisters less than 1 cm in diameter should be left intact, with the area surrounding the blister irrigated at least once per day followed by application of a topical antibiotic. A petrolatum gauze bandage can be put in place over these unbroken blisters, if desired. Any such dressings should be changed every 3 to 4 days.

While these handbooks recommend the use of bacitracin and triple combination preparations following cleansing of deroofed blisters, they do not provide specific application guidelines. We feel that the use of these ointments should be limited to small wounds (less than 1% TBSA) and employed for very brief periods (3–5 days) because of their high capacity to provoke allergic cutaneous reactions. Likewise, the use of 10% mafenide acetate cream should be avoided because of the severe pain that it causes when applied to partial-thickness wounds and the possibility of metabolic derangements. Such problems are not encountered with the use of 5% mafenide acetate solution, which should be used instead of the 10% cream.

## STRATEGIES FOR THE DEVELOPMENT OF IMPROVED THERAPIES

Previous animal studies have shown that surgically aggressive approaches are needed to prevent or minimize significant cosmetic and functional deficits that result from deep HD injury. For the best outcome, deep dermal/full-thickness cutaneous HD injuries require full-thickness debridement followed by autologous split-thickness skin grafting.^45^,^83^ These surgically aggressive approaches to deep HD injuries resulted in the return of barrier function, skin color, and mechanical properties (hardness and elasticity) to near-normal levels within 15 days of treatment in a weanling pig model.^83^ To be successful, the skin grafts must be placed on a hemostatically secure wound bed, devoid of blood clots, debris, or necrotic tissue. The recipient bed must have an adequate blood supply to nourish the skin grafts, and the grafts must be protected from shearing forces, motion, and mechanical disruption. A variety of modalities is available and may be employed in achieving initial graft adherence and subsequent acceptance (“take”). These include sutures, surgical staples, fibrin glue, tie-over bolsters, compression dressings, and a variety of antishear dressing techniques. The choice of fixation and dressing technique is determined by the size and location of the wounds, and the experience and preferences of the surgeon.

Split-thickness skin grafting was used late in a few cases during the Iran-Iraq conflict where healing was particularly slow^12^ and for the late treatment of some poorly healing, deep injuries sustained in 1992 by a civilian who came across an unexploded artillery shell from the First World War.^72^ The *NATO Handbook on the Medical Aspects of NBC Defensive Operations* states that grafting has rarely been required in the past and when it was attempted, graft acceptance has been poor.^76^ Surgical details of the grafting procedures used are not readily available, and the procedures may not have been optimal. In contrast to this handbook, Graham et al^45^ noted equally high graft acceptance rates following either full-thickness sharp surgical tangential excision or laser debridement using a deep dermal/full-thickness HD injury model in weanling pigs. In thermal burns management, deep burns are grafted to promote timely wound closure and improve outcome with minimal cosmetic and functional deficits. The decision to graft is based on depth of injury. As with thermal burns, depth of HD injury should be accurately assessed before treatment begins. Reported long-term effects such as fragile skin and scarring likely indicate that injury depth was not accurately diagnosed and treatment was not sufficiently aggressive. As with deep thermal burns, deep HD injuries will require surgically aggressive approaches.

While past HD wound-healing research in swine has concentrated on deep dermal/full-thickness injuries, superficial (epidermis only) and superficial dermal injuries may have greater clinical relevance on the battlefield. Partial-thickness injuries will likely not require such surgically aggressive approaches (eg, split-thickness skin grafting). Treatment strategies for improved healing of partial-thickness cutaneous HD injury have recently been formulated by a working group of researchers and physicians at government laboratories in the United States and United Kingdom. The strategies are described below. Research is underway to experimentally support these strategies and determine which medical devices, supplies, and pharmaceuticals are most efficacious.

It is important to recognize that for any therapeutic regimen to be successful, a healthy immunological,^84^ nutritional/metabolic,^85^ and psychological^86^ status needs to be maintained. Infections and perturbations in organ function also need to be closely monitored and addressed as needed.

The ultimate goal is to determine the most efficacious treatment regimen to be applied in the clinical management of HD casualties. The ideal regimen should return damaged skin to optimal appearance and normal function in the shortest time. Improved treatment will result in a better cosmetic and functional outcome for the patient and will enable the casualty to return to normal activities sooner.

### The pig as an animal model for efficacy testing of candidate treatment regimens

Since human testing with vesicating agents such as HD is unethical, candidate treatment regimens need to be tested in an appropriate animal model. The animal model of choice is the pig, due to the similarities between human and porcine skin.^87^–^97^ The comparable histological characteristics of pig and human skin are similarities in epidermal thickness and composition, epidermal enzyme patterns, epidermal tissue turnover time, lipid content, character of keratinous proteins, pelage density and pattern of hair growth, dermal structure, deposition of subdermal fat, and general morphology.^90^–^92^ In addition, pig skin is antigenically closer to human skin than is rodent skin. A number of human antibodies have been shown to cross-react in pig skin.^98^ US Environmental Protection Agency guidelines for dermal exposure assessment state that the percutaneous absorption of many compounds in the pig is similar to that found in humans.^93^ Dick and Scott^90^ found that pig skin permeability to selected lipophilic penetrants was closer to that of human skin than was rat skin. Klain et al^94^ concluded that pig skin was a good model for human skin metabolic studies. Meyer et al^95^ concluded that among the domestic species, the pig provides the most suitable experimental model for dermatological research on humans. Results from studies of experimental treatments in porcine models of partial-thickness wound healing have correlated well with results of clinical studies.^91^ These findings suggest that the pig is a suitable research animal to use for predicting cutaneous effects of xenobiotics in humans. Some differences between human and porcine skin have been noted, however. Pigs lack eccrine sweat glands,^92^ although hair and sebaceous gland number and distribution are similar.^91^ Tubular apocrine glands are present and lie adjacent to hair follicles, and are more numerous than in humans.^92^ The subepidermal vascular network is less dense than it is in humans; however, the pattern of vascularization in the lower region corresponds to that found in humans.^92^ In addition, the permeability of pig skin to HD may be significantly higher than that of human skin under certain conditions.^96^

Pigs have been widely used in vesicant research.^10^,^11^,^38^,^45^,^83^,^96^–^109^ Weanling pigs have often been used for their ease of handling during lengthy wound healing studies. They are small enough to be easily placed under chemical fume hoods during agent exposures and have a large enough body surface to allow multiple, large (3-cm-diameter) lesions to be placed on either the dorsum or the ventral abdominal surface. While they do grow during wound healing studies, functional data (eg, microcutaneous blood flow, skin color) can be normalized to surrounding unaffected skin^83^ and morphometric data can be normalized to total body surface area^45^ to take this growth into account. The healing process appears to be optimal in young pigs.^92^ The healing rate is faster in Yorkshire piglets than in mature Yucatan miniature pigs,^91^ and young pigs are highly resistant to contamination and infection.^92^ Furthermore, pigs are amenable to habituation and can be trained to allow noninvasive biophysical skin measurements to be obtained from HD-exposed sites on the dorsum without the need for restraint or anesthesia.^110^ Finally, the use of pigs in cutaneous ulcer and burn wound research is supported by the US Food and Drug Administration.^111^ Thus, weanling pigs appear to be a suitable model for examining the efficacy of treatment regimens without significant interference caused by high infection rates or slow healing rates. However, they would not likely be the ideal model for studying wound colonization or infection.

While there is no common laboratory animal species, including the pig, that generates frank blisters, as do humans, HD has been noted to induce microblisters in pigs.^10^ This lack of frank blistering is thought to be the result of a diminished superficial dermal vascular plexus, a densely arranged dermis, and lack of loose areolar tissue that precludes intercellular fluid accumulation.^92^

### Immediate treatment of cutaneous sulfur mustard casualties

This section describes the recommendations of the US-UK working group for the immediate treatment of HD casualties. The uses of anti-inflammatory agents, antioxidants, and occlusive/semiocclusive dressings are discussed. The potential need for replacement fluid therapy and management of intact blisters are also addressed.

For those patients who are beginning to present with erythema or those who are in the latent period and suspect an exposure may have occurred, systemic administration of an anti-inflammatory agent will likely help to decrease the amount of damage ultimately induced. The pro-inflammatory mediators IL-1β, IL-6, IL-8, and TNF-α are released by normal human epidermal keratinocytes in culture on exposure to HD.^112^ Sulfur mustard has also been shown to provoke an edema response and release of IL-6 in 2 different mouse models.^113^ Sulfur mustard–induced inflammatory responses themselves likely contribute to the severity of the pathology, and numerous animal studies have shown the benefits of prophylactic or therapeutic use of anti-inflammatory agents.^43^,^69^–^71^ There are active programs researching a variety of nonsteroidal anti-inflammatory drugs (NSAIDs) administered topically or systemically, alone and in various combinations. Top candidates that have shown efficacy in a mouse ear model include indomethacin, fluphenazine dihydrochloride, olvanil, retro olvanil, octyl homovanillamide, and other analogs of capsaicin.^69^–^71^ It remains to be determined which NSAID (or combination), route of administration, length of administration, and dosing regimen is the most efficacious in preventing or ameliorating the effects of HD on skin. It is likely that administration for 2 to 5 days will be required for an NSAID. Topically delivered intracellular scavengers such as 4-methyl-2-mercaptopyridine-1-oxide^69^ and dimercaprol^69^,^71^ have proved effective in animal experiments in reducing the severity of HD-induced cutaneous injuries, and concurrent use of one of these agents with an NSAID may yield the best results (Dr William J. Smith, PhD, US Army Medical Research Institute of Chemical Defense, Aberdeen Proving Ground, Md, oral communication, 2004). (It should be noted that mouse skin is very thin and permeable. Any topical agent showing efficacy in a mouse model should also be tested in another animal model such as the weanling pig.) Corticosteroid anti-inflammatory agents such as hydrocortisone (given systemically or topically for cutaneous HD injuries)^70^,^71^ and dexamethasone (tested in vitro on primary alveolar macrophages and given topically for ocular HD injuries)^114^,^115^ also appear to be promising therapeutic agents. There are other topical steroidal anti-inflammatory agents of much greater potency that would likely be very efficacious if used early in the lesion development stage, such as betamethasone dipropionate, clobetasol propionate, and diflorasone diacetate. Superpotent (Class 1), potent (Class 2), and upper mid-strength (Class 3) topical corticosteroids should be tested for their efficacy in ameliorating HD-induced cutaneous injury.

As previously discussed, depletion of GSH and accumulation of endogenous oxidants and ultimate formation of potent oxidizing species (eg, toxic lipid peroxides) may be contributory factors in HD-induced cytotoxicity.^1^ Topically applied HD has been shown to negatively affect antioxidant enzymes in blood cells and body tissues of rats.^116^ Several antioxidants have been shown to protect liver and lung from oxidative damage following inhalation or percutaneous exposure to HD in a mouse model.^117^ It has been suggested that administration of antioxidants may be protective and useful.^118^ Thus, initial antioxidant treatment aimed at affecting the progression of lesions that is instituted during the erythema phase may prove to be of benefit. The effectiveness and role of the interruption of the inflammatory cascade by the inclusion of topical and systemic antioxidant agents as well as a determination of the optimal timing for such therapy are important and intriguing avenues for investigation.

Placement of an occlusive or semiocclusive dressing will likely prove to be helpful in promoting autolytic debridement and preventing desiccation. Debridement will play a central role in improving the healing of cutaneous HD lesions, and beginning the process early may be beneficial. How soon following exposure these dressings can be applied remains to be determined. While maintaining a moist environment has long been known to facilitate wound healing,^119^–^122^ caution needs to be observed since very early occlusion that builds up moisture levels in the skin will exacerbate the lesion. In addition, there is a period following exposure to sulfur mustard at which off-gassing of unbound HD occurs following a vapor^104^ or liquid exposure in weanling pigs (J. S. Graham et al, unpublished data, 2001). The existence of a reservoir of unbound HD in human skin was previously discussed. These studies have suggested that off-gassing can continue for 24 to 36 hours, given a large exposure. Limiting the escape of this unbound HD by using an occlusive dressing may exacerbate the lesion. Placement of any occlusive dressing should probably be postponed for at least 24 hours following exposure. Keeping clothing off the exposed area, thereby not allowing vapors to build up, may also be of benefit.

The potential need for replacement fluid therapy (RFT) and caution in avoiding overhydration was previously discussed. Requirements for RFT can be studied experimentally. A scientific study using an appropriate animal model needs to be conducted to determine whether body weight and percent TBSA affected should be taken into consideration when trying to determine the total volume of fluid required. It is also important to determine which fluids are most appropriate, what infusion rates are needed, and when to commence RFT. Using instrumented pigs, large areas of injury can be induced, followed by close monitoring over time of important physiological parameters. The effects of different RFT protocols can then be determined. To generate large TBSA injuries, exposures with partial-body vapor chambers can be used. Alternatively, liquid HD can be diluted to volumes greater than that allowed under current surety regulations and fast-wicking material used to spread the liquid out over a large area. Previous research has enumerated a number of stable vehicles that are suitable for cutaneous exposure with liquid HD.^108^

Management of casualties presenting with intact blisters varies according to the situation and level of care. Avoidance of infection is paramount so that the depth of injury does not increase. Small blisters should not be disrupted until deroofing can be done under controlled conditions. Larger blisters that are already flaccid may require deroofing or collapsing under sterile conditions. Current data does not permit recommendation of a single best approach. Once the necessary conditions and skills are reached, intervention should be more aggressive, with the dual goals of avoiding wound infection and optimizing recovery. As detailed below, aggressive management of the cutaneous HD injury, as opposed to that conducted on thermal or toxic epidermal necrolysis blisters, will require removal of the wound edges into normal-appearing skin along the periphery of the lesion and debridement of the base of the blister through the damaged BMZ into healthy dermis. Accurately determining depth of injury will influence this step. These patients will also require pain management and close observation for the systemic effects of HD exposure. Absence of thorough removal of damaged tissues will greatly slow healing and will enhance scarring and contracture in all but the most minor injuries.

For patients presenting with intact frank blisters, it may be beneficial to aspirate the blister fluid with a sterile needle and syringe and allow the roof of the blister to act as a sterile dressing until a physician can remove it. Reattachment of blister roofs has been noted to occur in the treatment of vitiligo via epidermal grafting using the tops of suction blisters^123^,^124^ and in experimental suction blisters in humans following aspiration of blister fluid.^125^ The roofs of HD blisters, however, are not expected to reattach to the blister floor owing to HD-induced damage to basal cells and BMZ components. Sloughing is expected to eventually occur. For patients presenting with ruptured HD-induced blisters, careful removal of the blister roof with scissors, application of an antibiotic ointment, and placement of a sterile dressing is warranted. For both of these scenarios, more complete debridement will be necessary for large lesions, as described later.

### Treatment of established partial-thickness lesions: overview of the approach

The remaining sections of this review describe the recommendations of the US-UK working group for the treatment of established partial-thickness cutaneous HD lesions. The general approach that is being taken is to perform adequate debridement of partial-thickness injuries, then treat the lesions like chronic cutaneous ulcers or partial-thickness thermal burns using contemporary medical approaches.

Before treatment can begin, the extent and severity of cutaneous HD injuries must be determined. Following assessment of injury and deroofing of frank blisters, adequate wound debridement needs to be performed, followed by 1 or more treatment adjuncts. Examples of adjuncts under consideration are dressings, growth factors, skin substitutes, topical nutritional support, and Vacuum Assisted Closure™ (V.A.C.®), Kinetic Concepts, Inc (KCI), San Antonio, Tex.

### Injury assessment

Before HD injuries can be appropriately treated, assessment of the injuries must be made. Total body surface area of the injuries should be established and depth of injury determined. Total body surface area can be determined using Wallace's Rule of Nines and the Lund and Browder chart for estimating burn severity, as previously discussed.^77^,^78^ Determination of injury depth is a much more challenging task. Accurate depth assessment is important because it dictates how aggressive treatment needs to be to minimize or prevent cosmetic and functional deficits.

In thermal burns, depth of injury is typically assessed by physical examination. Surface appearance, the pinprick test to assess pain, the “blanch-capillary return test” to evaluate microcirculation, and surface temperature difference between burned and unburned skin are often used in diagnosis of depth.^126^ Using these methods, diagnosing very superficial and very deep burns is relatively easy for the experienced burn surgeon. Burns of intermediate depth are often problematic in determining the need for grafting. Determining depth of HD injuries is more challenging. First, the full extent of cutaneous injury can take several days to manifest. Second, superficial appearances do not accurately predict depth of injury or need for grafting. While the presence of blisters in thermal burns is generally associated with superficial dermal injuries, blistering in HD injuries can occur in deep dermal/full-thickness injuries because of the unique nature of the agent and the unique progression of the injury.

Noninvasively examining cutaneous blood flow using available bioinstrumentation can greatly assist the physician in determining depth of injury. Laser Doppler perfusion imaging and indocyanine green fluorescence imaging may prove to be very valuable tools in prognosticating optimal wound healing of both thermal burns and cutaneous HD injuries.

**Figure 1:**
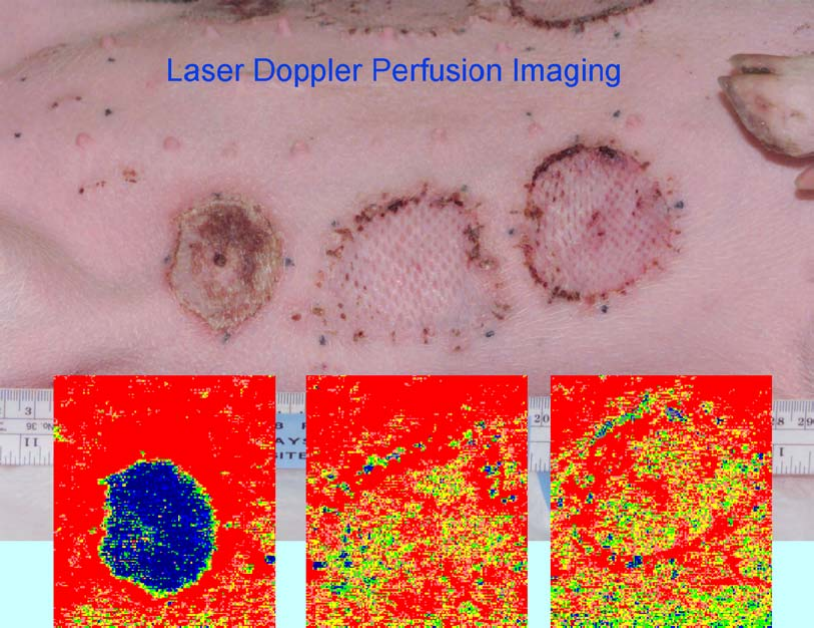
Laser Doppler perfusion imaging of deep dermal/full-thickness sulfur mustard injuries in a weanling pig model, 8 days after surgery. Sites were exposed to liquid sulfur mustard for 2 hours. Surgery was conducted 48 hours after agent exposure. The site on the left was untreated. The site in the center underwent full-thickness sharp surgical tangential excision followed by autologous split-thickness skin grafting. The site on the right underwent full-thickness laser debridement followed by grafting. Blue areas indicate low blood flux, red areas high blood flux and yellow/green areas intermediate blood flux. Analyses indicated poor blood flow in the untreated area and adequate blood flow in the grafted areas. Graft acceptance rates were equally high between both methods of debridement. Regardless of surgical approach, blood flux levels remained approximately 50% to 60% of normal tissue throughout a 36-day postsurgical observation period.^83^

Laser Doppler flowmetry and laser Doppler perfusion imaging (LDPI) have been used for prolonged, noninvasive monitoring of tissue viability and wound healing and for the assessment of peripheral vascular disease, inflammation, ischemia, reperfusion, skin graft acceptance (take), and burn depth.^127^–^142^ Laser Doppler perfusion imaging may prove useful in delineating the areas of HD damage that need to be debrided, thereby avoiding areas with sufficient blood flow. Brown et al^101^ found that laser Doppler perfusion images of vesicant vapor burns on the backs of swine correlated well with histopathological findings (thrombosis and necrosis of subepidermal capillaries) between 1 hour and 7 days postexposure and suggested that clinical management decision making for treatment of early vesicant burns could be aided by LDPI. Chilcott et al^102^ used several noninvasive bioengineering methods to monitor wound healing in a large white pig model for 7 days following exposure to HD and Lewisite vapors. They concluded that while reflectance colorimetry and TEWL measurements could provide quantitative, noninvasive methods for determining efficacy of candidate treatment regimens, neither is comparable to the prognostic capabilities of LDPI. Graham et al^83^ found LDPI to be useful in examining blood flow in grafted and ungrafted sites following treatment of deep dermal/full-thickness liquid HD injuries in a weanling swine model (Fig 1). Laser Doppler perfusion imaging is currently rather time consuming if there are multiple sites to be evaluated and/or large images to be collected at high resolution. The length of scanning procedures could be decreased by increasing scanning speed (thus decreasing flux resolution), decreasing the size of the scan area, and/or decreasing the number of lines scanned within the scanning area (scan resolution). Improvements in the technology that will speed up LDPI without compromising image resolution are being developed.

**Figure 2:**
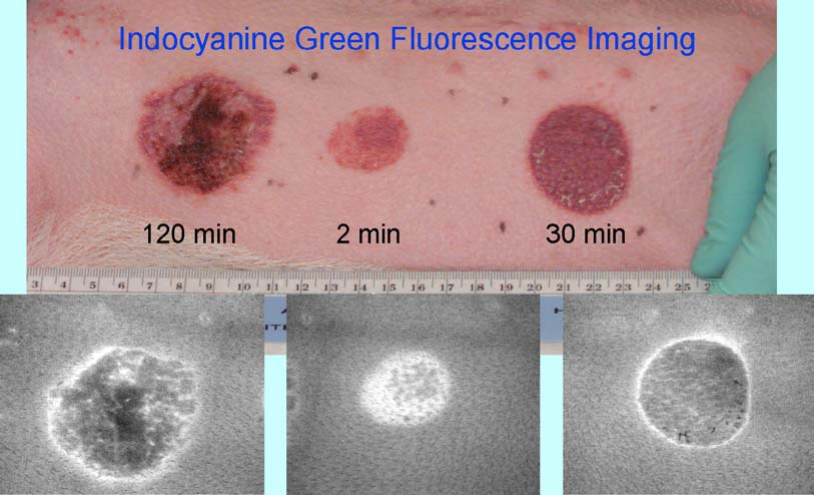
Indocyanine green fluorescence imaging of cutaneous sulfur mustard injuries in a weanling pig model, 48 hours after agent exposure. The fluorescence of intravenous indocyanine green has been shown to estimate thermal burn depth in small animals. Indocyanine green fluorescence is capable of distinguishing superficial and deep partial-thickness thermal burns from full-thickness burns. The fluorescence intensity of indocyanine green decreases exponentially with burn depth for thermal burns of similar age. Indocyanine green is a nontoxic and rapidly excreted tricarbocyanine dye that is strongly bound to serum proteins and leaks from patent vessels after an injury-induced increase in vessel permeability. Mean fluorescence levels within superficial injuries are much greater than those in surrounding normal tissue. In deep injuries where blood vessels are occluded, very little dye leaks into the extravascular space; hence mean fluorescence levels within deep injuries are much less than those in surrounding normal tissue. Shown here on the left, a deep dermal/full-thickness injury was generated after exposure to liquid sulfur mustard for 120 minutes, showing very dark patches in the center of the injury, surrounded by a ring of hyperemia. The center lesion is a superficial dermal injury that was generated after a 2-min exposure to sulfur mustard, showing a significant amount of fluorescence indicative of patent blood vessels. The lesion on the right is of intermediate severity. (J. S. Graham et al, unpublished data, 1999)

Indocyanine green fluorescence imaging has also shown promise in determining burn depth based on microcutaneous blood flow. It is a minimally invasive procedure that requires the placement of an intravenous line. Indocyanine green (ICG) is US Food and Drug Administration–approved for use in humans to determine cardiac output, hepatic function and blood flow, and for ophthalmic angiography. The fluorescence of intravenous ICG has been shown to estimate burn depth in small animals.^143^ In contrast to fluorescein fluorescence,^144^ ICG fluorescence is capable of distinguishing superficial and deep partial-thickness burns from full-thickness burns. The fluorescence intensity of ICG decreases exponentially with burn depth for burns of similar age.^145^ Indocyanine green fluorescence was used to estimate burn depth in a porcine model.^146^ An imaging system with a diagnostic algorithm was developed at the Wellman Laboratories of Photomedicine, Boston, Mass, that accurately diagnosed burns that healed within 21 days with minimal scarring from those that took longer to heal by secondary means. Measurements were made on burns created 2, 24, 48, and 72 hours prior to imaging. The algorithm was shown to be dependent on the age of the burn but not on location. This technology showed promise in plastic surgical applications^147^,^148^ and for accurate determination of thermal burn depth in humans.^147^,^149^ Indocyanine green fluorescence imaging also shows promise in diagnosing depth of HD injury (Fig 2; J. S. Graham et al, unpublished data, 1999). The advantage that this technology has over LDPI is the speed of image capture. Multiple images over large areas can be captured in a relatively short period of time. Images are typically collected 5 to 10 minutes after ICG injection to allow uptake and distribution. The dye is then excited (eg, 780 nm) and the resultant fluorescence emission (eg, 825 nm) immediately captured and saved by a computer and analyzed for burn-to-normal skin fluorescence ratio. Indocyanine green binds strongly to plasma globulins, limiting both extravasation within burn-injured vascular epithelia and extravascular transport to areas nearby.^145^ Large signals are thought to be the result of vasodilation and hyperemia, and smaller signals are thought to be attributable to vascular occlusion and edema.^143^,^145^ With this technique, live streaming video can also be captured immediately after injection, allowing the physician to watch the dye flowing through viable tissue and around nonviable tissue in real time.

It is of fundamental importance that any noninvasive or minimally invasive technique used to assess lesion severity be fully validated in order that results may be correctly interpreted. For example, a recent study has demonstrated that TEWL may not necessarily correlate with skin “barrier function.”^150^

### Debridement

Experimental approaches to vesicant wound debridement have included powered dermabrasion,^99^,^106^,^107^ sharp surgical excision,^45^,^83^,^151^ laser debridement,^45^,^83^,^100^,^107^,^109^,^152^ and enzymatic debridement.^152^

Powered dermabrasion has been shown to speed up the reepithelialization process of cutaneous HD injuries.^99^,^106^,^107^ Kjellstrom et al^151^ found sharp surgical excision with primary suturing of the skin defect to be effective in decreasing healing time of HD vapor lesions in guinea pigs. Powered dermabrasion, pulsed CO_2_ laser ablation, and erbium:yttrium-aluminum-garnet (Er:YAG) laser ablation have been shown to accelerate the rate of healing of full-thickness cutaneous Lewisite vapor burns in swine without the need for split-thickness skin grafting.^107^,^109^ Eldad et al^152^ found that excimer laser ablation and debrase (Debridase, MediWound Ltd, Yavne, Israel) enzymatic debridement were efficacious in improving the healing of partial-thickness nitrogen mustard burns in a guinea pig model.

Because of positive results achieved by laser and enzymatic debridement of vesicant injuries, our future research efforts will concentrate on the use of usthese methods to debride partial-thickness cutaneous HD injuries prior to the application of treatment adjuncts. The treatment regimen that is found to be most efficacious in the pig will ultimately be recommended for use in treating human casualties. Because humans form frank blisters, unlike the pig, these regimens would follow deroofing of any frank blisters present.

## LASER DEBRIDEMENT OF CUTANEOUS VESICANT WOUNDS

Laser debridement of cutaneous vesicant wounds has proven to be an effective method of improving the rate of wound healing in pig models. Graham et al^100^ showed that viability, thickness, and organization of the epidermis were all significantly improved by partial-thickness pulsed CO_2_ laser debridement of small, mild to moderately severe cutaneous HD vapor injuries. Laser debridement followed by skin grafting was as efficacious in improving the wound healing of deep HD burns as sharp surgical tangential excision followed by grafting (the gold standard in human deep dermal/full-thickness thermal burns medicine).^45^,^83^ Middermal debridement by sharp excision or laser ablation without grafting produced less desirable results but was better than no treatment.^45^,^83^ A 4-fold improvement in reepithelialization of Lewisite injuries was achieved at 1 week following laser dermabrasion, with almost 100% reepithelialization by 3 weeks.^109^ It is not apparent why these full-thickness Lewisite injuries (10 cm^2^) did not require grafting, as did HD injuries (12.6 cm^2^)^45^,^83^ or as would a full-thickness thermal burn. There are differences in biochemical action and rates of spontaneous reepithelialization between Lewisite and HD injuries.^109^ Further studies need to be conducted to fully examine the comparative healing of deep Lewisite, HD, and thermal injuries.

Laser debridement offers additional benefits including hemostatic control during surgery, minimal risk of exposure to aerosolized pathogens, and time efficiency. Another major advantage to the use of lasers is the ability to control the amount of normal perilesional skin that is removed. Eldad et al^152^ noted that it is technically difficult to control the amount of tissue to be removed by surgical tangential excision and that laser ablation of nitrogen mustard burns in a guinea pig model enabled both controlling the amount of tissue to be removed and minimizing blood loss. Minimizing the amount of tissue removed will be of a cosmetic benefit to the patient.

### Types of lasers available

Pulsed CO_2_ lasers have been used for a variety of dermatological applications, including skin resurfacing,^153^–^155^ excision of burn eschar,^156^–^159^ preparation of adequate graft beds,^160^ and conservative ablation of skin lesions.^161^ They are designed to promote rapid healing by minimizing laser-induced residual thermal damage,^153^,^154^,^160^,^162^–^166^ and offer precise, micrometer-depth removal of tissue.^166^ They vaporize tissue rapidly and efficiently, with minimal blood loss.^159^,^166^,^167^ However, because of the low average power available from a pulsed laser system, these lasers may be inefficient while performing full-thickness debridement of deep burns or while debriding burns covering large body surface areas. Continuous wave (cw) lasers can be quite efficient in removing tissue but tend to create significant amounts of thermal damage, sometimes creating more damage than the initial burn being treated. Domankevitz and Nishioka^167^ demonstrated that under appropriate conditions, a scanned cw CO_2_ laser could ablate tissue with a zone of residual thermal injury less than 200 μm, making it useful for cutaneous surgery and debridement of burn wounds prior to skin grafting. Residual thermal damage of cw lasers can be minimized if the laser is scanned over the surface rapidly enough that the amount of time the laser spends on any 1 point mimics a short laser pulse.^167^,^168^ Use of such lasers has proven efficacious in pigs^45^,^169^ and humans.^170^ Glatter et al^169^ usused a prototype cw CO_2_ laser in a thermal burn model in pigs and found that long-term scarring, based on Vancouver scar assessments, was equivalent at 6 months postsurgery in both laser-ablated + grafted and sharply excised + grafted burns. In addition, they noted no significant difference in engraftment rates between the 2 methods of debridement. Graham et al^45^ had similar success in engraftment rates using this same laser to treat HD injuries in pigs. In an initial clinical trial, Sheridan et al^170^ used a similar, commercially available system to perform full-thickness ablations of thermal burns in children. They found that no bleeding occurred in laser-ablated sites, that engraftment rates for both laser-ablated sites and sharply excised sites were equally high, and that there were no significant difference in Vancouver scar scores at an average follow-up of 32.0 ± 5.2 weeks.

There are a number of lasers manufactured in the United States, Canada, and Europe that could be considered for routine debridement of vesicant injuries. Acland and Barlow^171^ have provided a review on the current uses of lasers in dermatological practice and a list of the types of lasers used for specific procedures. They list CO_2_ and Er:YAG lasers as being the most appropriate for cutaneous resurfacing. While rapid-scanning, high-powered cw CO_2_ lasers would provide time-efficient ablation of damaged tissue,^45^ they are no longer commercially available. Pulsed CO_2_ lasers such as the UltraPulse (Lumenis Inc, Santa Clara, Calif) are in common use in dermatology and plastic surgery and have proved effective in improving wound healing of cutaneous vesicant injuries.^100^,^109^ Er:YAG lasers are also commercially available and have been used for a wide variety of procedures, ranging from facial resurfacing to burn debridement.^171^–^175^ They have been shown to be particularly useful in the debridement of partial-thickness burns^175^ and in the management of deep Lewisite injuries.^109^ Unlike the Gaussian beam profiles created by CO_2_ lasers, Er:YAG laser beams tend to be uniform and produce uniform depths of ablation.^175^ One commercially available unit, the Sciton PROFILE (Sciton Inc, Palo Alto, Calif), can be configured as a high-powered, dual-mode long-pulse Er:YAG laser that allows independent control of both depth of coagulation and depth of ablation. This versatility would be very advantageous to any clinic or hospital that treats burns and a variety of dermatological disorders. Er:YAG lasers will play a central role in future HD wound healing studies at our facilities.

## ALTERNATIVE METHODS OF DEBRIDEMENT UNDER CONSIDERATION

There are alternatives to using a laser to debride vesicant injuries. Sharp surgical tangential excisions and powered dermabrasion have proved effective.^45^,^99^,^106^,^107^,^152^ Curettage, cryotherapy, larval therapy, and enzymatic debridement may be effective, lower-cost alternatives.

Sharp surgical tangential excisions have been effective in the treatment of sulfur mustard^45^ and nitrogen mustard^152^ injuries. Powered dermabrasion has been shown to speed up the reepithelialization process of cutaneous sulfur mustard^99^,^106^ and Lewisite^107^ injuries. There are drawbacks with this method, however, including lack of uniform depth control and risk of aerosolizing pathogens.

Scraping, using dermal curettes, may be a viable option for removing desiccated material from the surface of the skin. Cryotherapy using ice, liquid nitrogen, or Peltier coolers may also be efficacious if applied early. The therapeutic effects of cooling pig skin soon after exposure to HD vapor has recently been reported.^176^ It may be possible to superficially freeze HD lesions before they proceed to vesication, thereby retarding the activity of the HD and progression of the lesion. The frozen tissue could then be removed using appropriate means, and the wound bed dressed with an antibiotic and sterile dressing until healed. Caution would have to be observed in the length of time the cooling agent is in direct contact with the skin and its potential to induce hypopigmentation taken into consideration. Finally, the use of larval therapy (maggots), while unconventional, has undergone a renaissance in the past few years and has proven to be very effective in debriding and improving the healing rate of hard-to-heal wounds (eg, chronic leg and foot ulcers).^177^–^184^ The success of this approach may warrant study as a possible treatment of small TBSA sulfur mustard injuries.

A final alternative under consideration for debridement of HD injuries is enzymatic debridement. These enzymes are categorized as proteolytics, fibrinolytics, and collagenases, and are designed to dissolve necrotic tissue from wounds.^185^ They are often used to debride chronic wounds (eg, decubitus ulcers, venous stasis ulcers, arterial insufficiency ulcers, diabetic foot ulcers). Many have been found to be safe and effective in removing devitalized tissue and accelerating healing in burns.^186^–^193^ Any burn eschar present is typically cross-hatched to allow the agent to penetrate into the wound. Other agents, such as the bacterial proteolytic enzymes streptokinase and streptodornase, have given disappointing results in deep burns because they do not break down the collagen that separates vital from nonvital tissue.^194^ Use of fibrinolysins may impair wound healing of HD lesions, as fibrin is an early matrix protein that is essential for wound healing. In addition, fibrinolysins are typically combined with deoxyribonuclease (DNase) and as such will also digest DNA in the dividing fibroblasts, which play a role in healing.^185^ Some effective enzymes have produced better results than others, with enzyme concentration, skin moisture level, and the presence of certain antibacterial agents affecting results. Secondary dressings are needed to keep the wound moist and to allow these agents to work.^185^ Klasen^194^ offers an excellent review of the use of enzymatic debridement agents in burns. The most popular and effective agents on the market today are collagenases (eg, Collagenase Santyl ointment, Ross Products Division, Abbott Laboratories Inc, Columbus, Ohio) and papain/urea combinations (eg, Accuzyme and Panafil, Healthpoint Ltd, Fort Worth, Tex; and Gladase Papain-Urea Debriding Ointment, Smith & Nephew Inc, Largo, Fla). In addition, a promising proteolytic enzyme extracted from the stem of the pineapple plant is in Phase II clinical trials in the United States and Europe for the treatment of deep partial- and full-thickness burns (Debrase Gel Dressing, MediWound Ltd, Yavne, Israel). Enzymatic debridement of HD injuries is a promising and cheaper alternative to laser debridement, albeit more time consuming. Research is planned for determining which available enzymatic debridement product is most efficacious in debriding partial-thickness HD injuries. The specific application regimen, the time required to reach adequate debridement, and potential adverse effects (eg, conversion to a deeper injury, infection) need to be determined in an appropriate animal model.

Burn wound sepsis and bacteremias have been noted in burn patients undergoing enzymatic debridement.^185^,^194^ Concomitant use of a topical antibiotic that does not interfere with the action of the enzyme under study may be warranted as a preventative measure.

## EXTENT OF DEBRIDEMENT REQUIRED

In addition to vesication and death of epidermal keratinocytes, HD exposure results in sublethal damage to keratinocytes along the periphery of the gross lesion. Damage to the basement membrane zone and underlying collagen in the papillary dermis has also been noted. Deroofing frank blisters followed by timely removal of this adjacent and subjacent damage will likely improve the rate of reepithelialization.

Nonlethal damage is clearly noted at the periphery of cutaneous HD lesions and has been reported previously.^11^,^105^,^195^ Nikolsky's sign,^196^ characterized by separation and loss of the epidermis from the dermis when the skin is pressed with a sliding or twisting motion, has been demonstrated in weanling pig skin following HD vapor exposure.^11^,^195^ These weakened areas of the dermal-epidermal junction occurred along the periphery of the gross lesions and are indicative of sublethally damaged basal cells and/or altered proteins of extracellular matrices of the BMZ. Sublethally injured cells at the periphery of an HD lesion and in hair follicles and other adnexal structures may be partly responsible for the slow rate of reepithelialization seen in these injuries. Rice et al^106^ suggested that the level of damage to cellular DNA at the margins of HD lesions may be sufficient to delay or prevent effective replication of those keratinocytes. Removal of these sublethally damaged keratinocytes at the margins of the lesions by debridement beyond the visible borders of the lesion will likely speed up the reepithelialization process.

As previously discussed, HD induces damage to the BMZ at the level of the lamina lucida.^5^,^39^ The floor of the blister retains portions of the damaged BMZ and needs to be removed to provide an adequate scaffold over which keratinocytes feeding the reepithelialization process can migrate. Thus, at minimum, debridement needs to proceed down into the papillary dermis after removal of the blister roof. Beyond the BMZ, dermal collagen itself is affected by HD exposure and can itself impede the wound healing process.^97^,^106^,^197^ Brown and Rice^197^ reported coagulation and hypereosinophilia of the papillary dermis in Yucatan minipig skin 12 to 24 hours following saturated HD vapor exposure, with the deeper reticular dermis unaffected. Rice et al^106^ and Lindsay and Rice^97^ suggested that following exposure to HD, papillary dermal collagen is altered and may no longer function normally as a healthy scaffold over which epidermal cells can migrate.

The question of how deep to debride needs to be addressed. Ablative lasers that create less than 160 ± 60 μm of residual thermal damage permit optimal skin graft take and healing.^160^ Domankevitz and Nisioka^167^ concluded that lasers that induce residual thermal damage zones of less than 200 μm are useful for cutaneous surgery and burn wound debridement prior to skin grafting. Lam et al^109^ were able to improve wound healing of full-thickness cutaneous Lewisite injuries in pigs by partial-thickness laser debridement. Graham et al^45^ were also able to improve wound healing of deep cutaneous HD injuries in pigs by partial-thickness debridement without grafting, albeit not to the extent attained by full-thickness debridement followed by grafting. These studies thus indicate that retaining some amount of damaged dermal tissue does not significantly impede wound healing. Complete debridement of partial-thickness injury, therefore, will likely not be required. Debridement of partial-thickness HD injury into the papillary dermis or upper reticular dermis will likely be adequate.

### Dressings

Following wound debridement of HD injuries, an appropriate dressing will be needed to promote moist wound healing. Beneficial effects of such dressings include prevention of tissue dehydration and cell death, accelerated angiogenesis, increased breakdown of dead tissue and fibrin (eg, pericapillary fibrin cuffs), significant reduction in pain, and potentiation of the interaction of growth factors with their target cells.^122^ Helfman et al^119^ and Singhal et al^185^ have provided overviews of the various types of occlusive and semiocclusive dressings. Hydrocolloids, hydrogels, foam dressings, alginates, and transparent film dressings are commercially available from a large number of manufacturers. As foam dressings and alginates are designed to control moderate to heavy exudates, they will likely not be needed for covering debrided cutaneous HD injuries. Silver impregnated dressing materials may be of great potential benefit in treating these wounds owing to their antimicrobial efficacy^198^–^200^ and demonstrated ability to enhance rates of reepithelialization.^201^,^202^ A number of these dressing materials are currently employed in burn and chronic wound care, while other more advanced silver dressings are in various stages of development. Thus, research on finding the most appropriate dressing during wound healing of these lesions should concentrate on hydrocolloids, hydrogels, thin films, and silver-impregnated dressings. Testing these products in a pig model should be adequate, since similar responses to occlusive and semiocclusive dressings on wound healing have been noted in pigs and humans.^203^

Weak attachment of the neoepidermis to the underlying dermis has been noted in human HD casualties^44^ and experimentally exposed weanling pigs (J. S. Graham et al, unpublished data, 2004). Once the lesions have fully reepithelialized, protective dressings may be needed to avoid or minimize damage as a result of friction with clothing or bedding.

### Growth factors

During cutaneous wound healing, growth factors play dominant roles in regulating cell proliferation, differentiation, and synthesis of extracellular matrix.^91^ Epidermal growth factor (EGF), transforming growth factor-β (TGF-β), platelet-derived growth factor (PDGF), insulin-like growth factor (IGF), keratinocyte growth factor (KGF), hepatocyte growth factor (HGF), granulocyte-macrophage colony-stimulating factor (GM-CSF), and fibroblast growth factors (FGFs) play important and critical roles in the healing of cutaneous wounds.^203^–^241^ Reviews of the effects of these growth factors on wound healing have been previously published.^203^–^208^ Improved wound healing has been reported for topical applications of EGF,^203^–^220^,^222^,^223^ PDGF,^203^–^208^,^214^,^219^,^224^–^229^ KGF,^206^,^207^,^231^–^233^ and IGF-I.^235^,^236^ There have been some negative reports on the effectiveness of EGF^221^ and KGF^230^ in improving wound healing of experimental split-thickness skin wounds in humans and esophagogastric anastomotic wounds in rats, respectively. Epidermal growth factor has been shown to improve the healing of graft donor sites,^210^ corneal burns,^217^,^220^ and cutaneous burns,^218^,^219^ whereas PDGF and KGF have been shown to improve the healing of burns^219^ and skin-grafted lesions.^224^,^233^

Human leptin is a 146–amino acid residue, nonglycosylated polypeptide involved in body weight regulation. It is released from white adipose tissue and exerts its effect via receptors in the hypothalamus. While not a growth factor per se but rather characterized as a satiety-regulating cytokine, leptin has been shown to be a potent mitogenic stimulus to keratinocytes during skin repair.^237^,^238^

Results from these reports suggest that topical application of healing-enhancing factors alone or in combination may be beneficial in improving the wound healing of cutaneous HD injuries following wound debridement. When to commence such treatment following agent exposure needs careful consideration and experimental testing. During the early phases of wound healing, chemokines and cytokines regulate the chemotaxis and activation of inflammatory cells, along with synthesis of proteases and protease inhibitors.^91^ Early application of growth factors would be ineffective in a milieu of proteases. Application of such growth factors or combinations thereof will likely require a delay of 3 to 7 days following HD exposure until the inflammatory response has subsided. Concomitant use of protease inhibitors or a dressing that binds matrix metalloproteases and protects growth factors (eg, PROMOGRAN* Matrix Wound Dressing, Johnson & Johnson Wound Management Worldwide, Somerville, NJ) may be necessary. A number of these growth factors are commercially available for efficacy testing in animal models (EGF, PDGF, leptin). Of these 3, only PDGF has been approved for human use by the US Food and Drug Administration. Recombinant human EGF (β-urogastrone) is available from Roche Diagnostics Corp, Indianapolis, Ind. Regranex Gel, a recombinant human PDGF-BB, is available from Johnson & Johnson Wound Management Worldwide, Somerville, NJ. Recombinant human leptin is available from R&D Systems Inc, Minneapolis, Minn. Amgen Inc (Thousand Oaks, Calif) has recently completed Phase III clinical trials of a recombinant human KGF (Palifermin) that significantly reduces the duration and incidence of radiation- and chemotherapy-induced oral mucositis.

In addition to these growth factors, topical application of extracellular matrix components such as fibronectin, which supports fibroblast, keratinocyte, and endothelial cell adhesion and movement,^242^,^243^ may be of benefit following wound debridement. Fibronectin can serve as a template for collagen deposition^242^ and is a key component of the provisional matrix during wound repair.^243^ Exogenous application of intact fibronectin has been shown to be beneficial in helping to close human skin and corneal wounds.^244^ The ability of keratinocytes to spread on fibronectin may require the presence of serum or epibolin/vitronectin.^244^ Addition of another adhesive glycoprotein, laminin, may actually be detrimental to the reepithelialization process. When human keratinocytes are placed in apposition with collagen, they attach and begin migrating.^244^ This migration is inhibited by the addition of laminin, which acts as a major cell adhesion factor for keratinocytes. Laminin 5 has been shown to inhibit human keratinocyte migration and strongly promotes keratinocyte attachment.^244^ It is believed to anchor the keratinocytes to the substratum, via α_3_β_1_-integrin receptors.^244^

### Skin substitutes

Skin substitutes may provide an excellent temporary wound dressing for debrided HD injuries. Permanent wound closure can only be achieved by spontaneous reepithelialization or by the provision of autologous skin by means of skin grafting. The use of skin substitutes to temporarily restore the multiple functions of normal skin may be of substantial benefit in the management of cutaneous HD injuries.

For a skin substitute application to be successful, the same conditions required for successful autograft “take” must be created and maintained. The selection of the most suitable and effective temporary skin substitute will require a critical assessment of its comparative attributes when applied to HD wounds as well as the issues of cost, ease of use, availability, and consistency of results. Skin substitutes are widely used in human thermal burns management and can be (1) temporary or permanent; (2) epidermal, dermal, or composite; and (3) biologic or synthetic.^245^–^253^ They have also been shown to be effective in speeding up time to closure of chronic leg and foot ulcers,^254^–^269^ surgical excision sites,^269^ and partial-thickness donor sites.^269^ They may be a source of growth factors and are generally semiocclusive in nature. They can provide barrier function; add tensile strength to the wound; are generally flexible and pliable; markedly reduce pain, inflammation, and drainage; and provide a moist wound healing environment. They do not control deep bacterial infections; can seal bacteria in; and, being a biologic, they can transmit infection. Hence, the wound surface must not be infected for application of a skin substitute. A number of skin substitutes are available on the market and should be tested for their efficacy in improving wound healing of cutaneous HD injuries. Marketed products currently under consideration include (1) living bilayered skin substitutes (APLIGRAF [Organogenesis Inc, Canton, Mass], designed for the treatment of venous leg ulcers and diabetic foot ulcers; and OrCel [Ortec International Inc, New York], designed for the management of split-thickness donor site wounds and for the treatment of epidermolysis bullosa), (2) bilayered composites consisting of a synthetic epidermal analog and a biologic (collagen-based) dermal analog (TransCyte [Smith & Nephew Inc, Hull, United Kingdom] and Biobrane [Bertek Pharmaceuticals Inc, Morgantown, Wva], both designed for partial-thickness wounds), (3) complex weaves of biopolymers that produce a thin protective membrane (Silon-TSR [Bio Med Sciences Inc, Allentown, Pa], designed for use on partial-thickness burns, donor sites, and laser-resurfaced skin), and (4) acellular dermal matrices designed to aid in the natural healing of partial-thickness injuries of limited depth (SkinTemp, BioCore Medical Technologies Inc, Silver Spring, Md). Permanent skin substitutes that are designed for treating deep injuries and require application of a thin epithelial autograft will likely be inappropriate for use in treating partial-thickness HD injuries (eg, AlloDerm [LifeCell Corporation, Blanchburg, NJ] and INTEGRA dermal regeneration template [Integra LifeSciences Holdings Corporation, Plainsboro, NJ]).

Cryopreserved and glycerol-preserved cadaver skin has been used as a temporary dressing in the treatment of burns for a number of years.^270^–^277^ Similarly, xenografts from a variety of animal species, especially pig, have been used as temporary cover to treat burns.^278^–^280^ While pig skin is antigenic and would ultimately get sloughed, portions of the dermis may become incorporated in a healed wound and elicit an unwanted granular response.^278^

Cultured epithelial allografts and autografts have been used for about 2 decades as a treatment for chronic ulcers and thermal burns.^248^,^281^–^312^ Keratinocytes can be harvested from skin biopsies and grown to confluence by the method originally described by Rheinwald and Green.^313^ Large amounts of stratifying epidermis can thus be grown in the laboratory in short periods of time and used to restore defects in the epidermis.^314^ Such grafts can be used immediately or cryopreserved and used at a later date. In addition to their usefulness in improving the healing of deep ulcers and burns, they have shown efficacy in improving the rate of reepithelialization of partial-thickness burns^286^,^290^,^300^ and split-thickness skin graft donor sites.^289^,^291^,^307^ There is no evidence that cultured allografts survive permanently on the wound bed.^289^ Kaawach et al^286^ showed that allografted cells were not present between 8 and 100 days postgrafting and suggested that the newly formed epithelium was of host origin. Cultured keratinocyte allografts speed healing by providing cover and producing growth factors and extracellular matrix proteins.^291^ Because these coverings can be produced in large quantities and would thus be more readily available than cadaver skin, their application in the treatment of debrided partial-thickness HD injuries should be considered. Cultured epidermal autografts (CEAs) would be safer to use, from the perspective of disease transmission, and would not require donor-screening procedures. They do, however, require small punch biopsies to be collected from the patient and a lag time of about 2 weeks to grow the graft material. Several laboratories in the United States perform this service for their local burn centers (eg, Living Skin Bank, University Hospital, SUNY, Stony Brook, NY). Genzyme Corporation (Cambridge, Mass) has shown that CEA (Epicel) can be commercially produced. Despite their theoretical usefulness, CEAs are rather limited in their clinical effectiveness because they are unable to withstand even very low levels of bacterial wound contamination and do not provide a durable epithelial surface. Wounds covered by this modality are unstable and are subject to frequent epithelial disruption as a result of minor mechanical trauma. Durability has been increased by placing the CEA on a scaffolding of widely meshed autograft.^315^ Alternatively, CEA placed over deepithelialized allograft (ie, engrafted allodermis) has also proved successful.^284^

Finally, application of keratinocytes in suspension has shown to improve epidermal wound healing in pig^316^,^317^ and mouse^318^,^319^ models. Reconstitution of the dermal-epidermal junction was significantly enhanced in an athymic mouse model by suspending the cells in a fibrin-glue matrix.^319^ Using a pig model, Currie et al^320^ recently compared the effects of keratinocyte cell sprays with and without fibrin glue. No differences in mean epithelial area or quality of epithelium were noted at 3 weeks. Keratinocyte suspension technology shows promise in that it does not require the length of time necessary to produce cultured epidermal sheets. Use of this technology has proven efficacious in the treatment of thermal burns in humans.^321^ A small biopsy is collected and the cells cultured and expanded in a clinical laboratory, then placed into a syringe-like spraying mechanism and sprayed onto the wound 2 to 5 days following biopsy. This technology is currently available (CellSpray and CellSpray XP, Clinical Cell Culture, Bentley, Western Australia). These products are designed for use in partial, deep partial, and full-thickness burns, donor sites, scar treatment, chronic ulcers, pigment loss, and cosmetic skin rejuvenation following laser resurfacing, dermabrasion, or chemical peels. A similar spray-on product in development that delivers allogeneic keratinocytes, fibroblasts, and fibrin to wounds has recently shown positive results in Phase II trials in Europe and the United States (Allox, IsoTis OrthoBiologics, Irvine, Calif). An innovative medical device (ReCell, Clinical Cell Culture, Bentley, Western Australia) has been developed that will allow rapid harvesting of cells from a thin split-thickness biopsy followed by spray application onto wounds within 30 minutes of collecting the biopsy, without the need of culturing the keratinocytes in a clinical laboratory. This single-use device is designed for injuries up to 2% TBSA. It may prove beneficial in the treatment of small TBSA HD injuries and is worthy of laboratory investigation.

### Topical nutritional support

Boyce et al^322^ noted that application of topical nutrients supports keratinocyte viability during graft vascularization of cultured skin substitutes and inhibits wound contraction. There are a large number of “cosmeceutical” products on the market designed to enhance the appearance, feel, flexibility, and function of skin by supplying moisturizing and nutritive substances. Amino-Plex Spray (biO_2_ Cosmeceuticals International Inc, Beverly Hills, Calif) is such a product that is designed to increase oxygen in cells, stimulate ATP synthesis, improve glucose transportation, stimulate collagen formation, and promote angiogenesis. It is a mixture of over 100 low-molecular-weight ingredients, including amino acids, trace minerals, nucleotides, nucleosides, oligopeptides, electrolytes, glycosaminoglycans, and glycolipids. According to the company, this product has been shown to reduce irritation and improve results in laser resurfacing, chemical peels, microdermabrasion, hair transplantation, and hair removal. A new product (Oxy-Mist, biO_2_ Cosmeceuticals International Inc, Beverly Hills, Calif) combines the ingredients of Amino-Plex Spray with micellized vitamin E and sterile mineralized water and uses medical-grade oxygen as the delivery source. The manufacturer reports that used after facial resurfacing with a pulsed CO_2_ or erbium laser, Oxy-Mist has been clinically shown to accelerate reepithelialization, minimize pain, and decrease the period of postlaser erythema. The oxygen itself likely contributed to the improved healing noted, as both hyperbaric^323^ and topical^324^ oxygen therapies have been shown to facilitate wound healing. Because of its reported benefits in dermatology following laser resurfacing procedures, it would be advantageous to determine whether these topical nutritional products will improve the healing of debrided HD injuries.

### Vacuum assisted closure

Application of topical negative pressure in the management of chronic wounds and burns has gained popularity in the last 5 years.^325^–^332^ Also known as Vacuum Assisted Closure (V.A.C.), the procedure involves placing an open-cell foam into the wound bed (cut to conform to the shape of the wound), sealing it with an adhesive drape and applying subatmospheric pressure (125 mm Hg below ambient) that is transmitted via an evacuation tube by a computerized vacuum pump.^326^,^327^ The procedure is becoming widely used for the closure of chronic wounds such as stage III and IV pressure ulcers; venous, arterial, and neuropathic ulcers; and subacute and acute wounds such as dehisced incisions, split-thickness meshed skin grafts, and muscle flaps.^326^,^327^ V.A.C. is also gaining popularity in the management of complex orthopedic wounds.^329^,^330^ This methodology increases local blood perfusion and nutrient delivery to the wound, accelerates the rate of granulation tissue formation, and decreases wound tissue bacterial levels.^326^,^327^ Per the manufacturer's recommendations, wounds must be debrided of all necrotic tissue prior to application of V.A.C., and it is contraindicated with the presence of nonenteric and unexplored fistulas, osteomyelitis (untreated), exposed organs or blood vessels, or malignancy in or around the wound. The dressings are typically changed every 1 to 4 days until wound closure. V.A.C. has been shown to be effective in preventing progression of partial-thickness burns to a deeper injury in a swine model,^333^ likely the result of helping to deliver oxygen and nutrients to the zone of stasis. The method has also been shown to increase the rate of skin graft donor site reepithelialization in pigs and humans^331^ and is a safe and effective method for securing split-thickness skin grafts, providing improved graft survival.^332^ Following debridement of partial-thickness HD injuries, V.A.C. may prove efficacious in significantly speeding the reepithelialization process in these lesions. Recently, the US Food and Drug Administration approved the use of V.A.C. in treating partial-thickness burns. The expedited closure of HD wounds by means of a mechanical force is an area that merits further consideration and investigation. Several V.A.C. therapy systems are available from Kinetic Concepts Inc, San Antonio, Tex. One lightweight portable system is available for ambulatory care.

### Use of noninvasive bioengineering methods to assess treatment efficacy

During efficacy testing of candidate treatment regimens, it is important to examine a number of parameters besides reepithelialization. While coverage of the wound by a new epithelium is important, there are a number of other skin characteristics that are important from a functional and cosmetic point of view. Surface contour and general appearance, epidermal hydration, epidermal barrier function, pH, mechanical properties, cutaneous blood flow, transcutaneous oxygen tension, neural supply/sensory function, and hair growth are all important characteristics that bear examination. While routine histopathology, immunohistochemistry, and electron microscopy are all valued tools in determining morphology and understanding the pathophysiology of HD wound development and healing, they do not directly measure physiological parameters or function. For these, a variety of noninvasive bioengineering methods are available. In support of HD wound healing research, laboratories in the United States and United Kingdom have used reflectance colorimetry to evaluate erythema, skin hue, chroma, and lightness^11^,^27^,^83^,^100^,^102^; LDPI to examine cutaneous blood flow, depth of injury, neovascularization, and skin graft viability^83^,^101^; torsional ballistometry to evaluate the mechanical properties of skin firmness and elasticity^83^; evaporimetry to examine transepidermal water loss as a way to evaluate skin hydration/barrier function^83^,^102^; 2-dimensional and 3-dimensional high-frequency (20-MHz) ultrasound to examine edema formation^11^ and scar tissue thickness (J. S. Graham et al, unpublished data, 2002); and image analysis to evaluate wound size, shape morphometry, and wound contraction.^45^,^100^ While not yet used in HD wound healing research efforts, instrumentation is also available for evaluating surface contour, pH, and sensory function.

## SUMMARY

The toxicity of sulfur mustard has been widely described.^1^,^3^–^16^ Cutaneous HD injuries can take several months to heal, may necessitate lengthy hospitalizations, and can result in significant cosmetic and/or functional deficits. There are currently no standardized or optimized methods of casualty management that prevent or minimize deficits and provide for speedy wound healing.

Research laboratories in the United States, United Kingdom, and Canada have developed concepts for medical countermeasures to vesicant agents. The initial step in protecting a person from the deleterious effects of HD is to eliminate contact with the agent. Protective gear and topical skin protectants have been designed for this purpose. Should the agent come in contact with the skin, it needs to be removed within 2 minutes to fully prevent damage.^15^ Decontamination is generally performed by physical removal. Sulfur mustard is not painful on contact and the exposed person may not be aware of the exposure until symptoms begin to appear after a latent period. Pharmacological approaches are being studied for their efficacy in minimizing or preventing damage. At this time, it seems clear that the earliest possible application of anti-inflammatory agents, including cold packs, topical and systemic steroids, and nonsteroidal anti-inflammatory drugs, is beneficial. Should HD come in contact with the skin, were decontamination not performed timely, and should pharmacological intervention be absent or ineffective, a chemical casualty will be produced that requires medical attention. Casualty management now comes into play. There are no antidotes to HD. Therapy therefore rests on management of symptoms and consequences of exposure with the intent to reduce long-term morbidity. Historically, blister aspiration and/or deroofing (epidermal removal), physical debridement, irrigation, topical antibiotics, and sterile dressings have been the main courses of action in the medical management of cutaneous HD injuries. New strategies to relieve symptoms, prevent infections, and promote healing have been formulated. Deep cutaneous HD injuries will require aggressive surgical intervention, including skin grafting, if cosmetic and functional deficits are to be avoided. Our future research efforts will concentrate on partial-thickness injury that will not require such aggressive approaches.

Assessment of the injuries must occur early in the process. Total body surface area of the injuries should be established and depth of injury determined. Laser Doppler perfusion imaging and ICG fluorescence imaging show promise in prognosticating optimal wound healing of HD injury on the basis of examination of microcutaneous blood flow. Following assessment of HD injury, adequate wound debridement needs to be performed. At the minimum, debridement needs to proceed into normal-appearing skin along the periphery of the lesion and down through the base of the blister (eg, damaged BMZ) into the papillary dermis. Debridement is then followed by 1 or more treatment adjuncts. Such adjuncts under consideration are dressings, growth factors, skin substitutes, topical nutritional support, and Vacuum Assisted Closure.

The ultimate goal is to determine the most efficacious treatment regimen to be applied in the clinical management of HD casualties. The ideal regimen should return damaged skin to optimal appearance and normal function in the shortest time. Improved treatment will result in a better cosmetic and functional outcome for the patient, and will enable the casualty to return to normal activities sooner.

## References

[1] Papirmeister B, Feister AJ, Robinson SI, Ford RD (1991). Medical Defense Against Mustard Gas: Toxic Mechanisms and Pharmacological Implications.

[2] United Nations Centre for Disarmament Affairs (1994). Disarmament: The Chemical Weapons Convention With Selective Index.

[3] Somani HD, Babu SR (1989). Toxicodynamics of sulfur mustard. Int J Clin Pharmacol Ther Toxicol.

[4] Petrali JP, Oglesby SB, Mills KR (1990). Ultrastructural correlates of sulfur mustard toxicity. J Toxicol-Cut & Ocular Toxicol.

[5] Petrali JP, Oglesby SB, Hamilton TA, Mills KR (1993). Ultrastructural pathology and immunohistochemistry of mustard gas lesion.

[6] Vogt RF, Dannenberg AM, Schofield BH, Hynes NA, Papirmeister B (1984). Pathogenesis of skin lesions caused by sulfur mustard. Fundam Appl Toxicol.

[7] Monteiro-Riviere NA, King JR, Riviere JE (1991). Mustard induced vesication in isolated perfused skin: biochemical, physiological and morphological studies.

[8] Monteiro-Riviere NA, Inman AO (1993). Histochemical localization of three basement membrane epitopes with sulfur mustard induced toxicity in porcine skin. Toxicologist.

[9] Zhang Z, Peters BP, Monteiro-Riviere NA (1995). Assessment of sulfur mustard interaction with basement membrane components. Cell Biol Toxicol.

[10] Mitcheltree LW, Mershon MM, Wall HG, Pulliam JD, Manthei JH (1989). Microblister formation in vesicant-exposed pig skin. J Toxicol Cutan Ocul Toxicol.

[11] Graham JS, Martin JL, Zallnick JE (1999). Assessment of cutaneous sulfur mustard injury in the weanling pig. Skin Res Technol.

[12] Mellor SG, Rice P, Cooper GJ (1991). Vesicant burns. Br J Plast Surg.

[13] Requena L, Requena C, Sanchez M (1988). Chemical warfare. Cutaneous lesions from mustard gas. J Am Acad Dermatol.

[14] Borak J, Sidell FR (1992). Agents of chemical warfare: sulfur mustard. Ann Emerg Med.

[15] Sidell FR, Urbanetti JS, Smith WJ, Hurst CG, Sidell FR, Takafuji ET, Franz DR (1997). Vesicants. Warfare, Weaponry and the Casualty-Medical Aspects of Chemical and Biological Warfare.

[16] Sidell FR, Hurst CG, Sidell FR, Takafuji ET, Franz DR (1997). Long-term health effects of nerve agents and mustard. Warfare, Weaponry and the Casualty - Medical Aspects of Chemical and Biological Warfare.

[17] Ghanei M, Aslani J, Khateri S, Hamadanizadeh K (2003). Public health status of the civil population of Sardasht 15 years following large-scale wartime exposure to sulfur mustard. J Burns Wounds [serial online].

[18] Khateri S, Ghanei M, Soroush MR, Haines D (2003). Effects of mustard gas exposure in pediatric patients: long-term health status of mustard-exposed children, 14 years after chemical bombardment of Sardasht. J Burns Wounds [serial online].

[19] Lin P, Bernstein IA, Vaughan FL (1994). Failure to observe a relationship between bis-(beta-chloroethyl) sulfide-induced NAD depletion and cytotoxicity in the rat keratinocyte culture. J Toxicol Environ Health.

[20] Mol MA, van der Schans GP, Lohman PH (1993). Quantification of sulfur mustard-induced DNA interstrand cross-links and single-strand breaks in cultured human epidermal keratinocytes. Mutat Res.

[21] Mol MA, van de Ruit AM, Kluivers AW (1989). NAD^+^ levels and glucose uptake of cultured human epidermal cells exposed to sulfur mustard. Toxicol Appl Pharmacol.

[22] Orrenius S, Nicotera P (1987). On the role of calcium in chemical toxicity. Arch Toxicol.

[23] Smith CN (1994). A Possible Initiating Role for Melanocytes in Sulphur Mustard Induced Skin Lesions.

[24] Brimfield AA, Zweig LM, Novak MJ, Maxwell DM (1998). In vitro oxidation of the hydrolysis product of sulfur mustard, 2,2′-thiobis-ethanol, by mammalian alcohol dehydrogenase. J Biochem Mol Toxicol.

[25] Papirmeister B (1994). Does apoptosis (programmed cell death) play a role in sulfur mustard injury?. Med Chem Defense.

[26] Kan RK, Pleva CM, Hamilton TA, Anderson DR, Petrali JP (2003). Sulfur mustard-induced apoptosis in hairless guinea pig skin. Toxicol Pathol.

[27] Braue EH, Mershon MM, Wade JV, Litchfield MR (1990). In vivo assessment of vesicant skin injury using a Minolta Chroma Meter. J Soc Cosmet Chem.

[28] Braue EH, Koplovitz I, Mitcheltree LW, Clayson ET, Litchfield MR, Bangledorf CR (1992). Characterization of the sulfur mustard vapor induced cutaneous lesions on hairless guinea pigs. Toxicol Methods.

[29] Dannenberg AM (1990). Sulfur Mustard (HD) Lesions in Organ-cultured Human Skin: Markers of Injury and Inflammatory Mediators. Final Report.

[30] Graham JS, Bryant MA, Braue EH (1994). Effect of sulfur mustard on mast cells in hairless guinea pig skin. J Toxicol Cutan Ocul Toxicol.

[31] Marlow DD, Mershon MM, Mitcheltree LW, Petrali JP, Jaax GP (1990). Evaluation of euthymic hairless guinea pigs [crl:IAF(HA)BR] as an animal model for vesicant injury. J Toxicol Cutan Ocul Toxicol.

[32] Mershon MM, Mitcheltree LW, Petrali JP, Braue EH, Wade JV (1990). Hairless guinea pig bioassay model for vesicant vapor exposures. Fundam Appl Toxicol.

[33] Moore KG, Schofield BH, Higuchi K (1986). Two sensitive *in vitro* monitors of chemical toxicity to human and animal skin (in short-term organ culture), I: paranuclear vacuolization in glycol methacrylate tissue sections, II: interference with [^14^C]leucine incorporation. J Toxicol Cutan Ocul Toxicol.

[34] Papirmeister B, Gross CL, Petrali JP, Hixson CJ (1984). Pathology produced by sulfur mustard in human skin grafts on athymic nude mice, I: gross and light microscopic changes. J Toxicol Cutan Ocul Toxicol.

[35] Yourick JJ, Clark CR, Mitcheltree LW (1991). Niacinamide pretreatment reduces microvesicle formation in sulfur mustard cutaneously exposed hairless guinea pigs. Fundam Appl Toxicol.

[36] Petrali JP, Oglesby SB, Justus TA (1991). Morphologic effects of sulfur mustard on a human skin equivalent. J Toxicol Cutan Ocul Toxicol.

[37] Petrali JP, Oglesby SB, Hamilton TA, Mills KR (1993). Comparative morphology of sulfur mustard effects in the hairless guinea pig and a human skin equivalent. J Submicrosc Cytol Pathol.

[38] King JR, Monteiro-Riviere NA (1990). Cutaneous toxicity of 2-chloroethyl methyl sulfide in isolated perfused porcine skin. Toxicol Appl Pharmacol.

[39] Petrali JP, Oglesby SB, Hamilton TA, Mills KR (1992). Ultrastructural pathology and immunohistochemistry of mustard gas lesion.

[40] Renshaw B (1946). Mechanisms in the production of cutaneous injuries by sulfur and nitrogen mustards. Chemical Warfare Agents and Related Problems.

[41] Chilcott RP, Jenner J, Carrick W, Hotchkiss SAM, Rice P (2000). In vitro human skin absorption of bis (2-chloroethyl)sulphide (sulphur mustard). J Appl Toxicol.

[42] Nagy HD, Golumbic C, Stein WH, Fruton JS, Bergmann M (1946). The penetration of vesicant vapors into human skin. J Gen Physiol.

[43] Smith WJ, Dunn MA (1991). Medical defense against blistering chemical warfare agents. Arch Dermatol.

[44] Willems JL (1989). Clinical management of mustard gas casualties. Ann Belg Med Mil.

[45] Graham JS, Schomacker KT, Glatter RD, Briscoe CM, Braue EH, Squibb KS (2002). Efficacy of laser debridement with autologous split-thickness skin grafting in promoting improved healing of deep cutaneous sulfur mustard burns. Burns.

[46] Pechura CM, Rall DP (1993). Veterans at Risk: The Health Effects of Mustard Gas and Lewisite.

[47] O'Hern MR, Dashiell TR, Tracy MF, Sidell FR, Takafuji ET, Franz DR (1997). Chemical defense equipment. Warfare, Weaponry and the Casualty—Medical Aspects of Chemical and Biological Warfare.

[48] Chilcott RP, Jenner J, Hotchkiss SA, Rice P (2002). Evaluation of barrier creams against sulphur mustard, I: in vitro studies using human skin. Skin Pharmacol Appl Skin Physiol.

[49] Braue EH (1999). Development of a reactive topical skin protectant. J Appl Toxicol.

[50] Snider TH, Matthews MC, Braue EH (1999). A model for assessing efficacy of topical skin protectants against sulfur mustard vapor using hairless guinea pigs. J Appl Toxicol.

[51] Hobson ST, Lehnert EK, Braue EH (2000). The U.S. Army reactive topical skin protectant (rTSP): challenges and successes. MRS Symp Ser CC: Hybrid Org Inorg Mater [serial online].

[52] Hobson ST, Braue EH (2001). Development of multifunctional perfluorinated polymer blends as an active barrier cream against chemical warfare agents. Polym Mater Sci Eng.

[53] Braue EH, Hobson ST, Miziolek AW, Karna S, Matthew JM (2005). Nanomaterials as active components in chemical warfare agent barrier creams. Defense Applications of Nanomaterials.

[54] Braue EH, Hobson ST, Govardhan C, Khalaf N (2002). Active topical skin protectants containing OPAA enzymes and CLECs.

[55] Braue EH, Mershon MM, Braue CR, Way RA (2002). Active topical skin protectants containing S-330.

[56] Braue EH, Hobson ST, White J, Bley R (2002). Active topical skin protectants using polyoxometallates.

[57] Braue EH, Hobson ST, Hill CL, Boring E, Rhule J (2002). Active topical skin protectants using polyoxometalates and/or coinage metal complexes.

[58] Braue EH, Hobson ST, Lehnert EK (2002). Active topical skin protectants.

[59] Hobson ST, Braue EH, Back D (2002). Active topical skin protectants using polymer coated metal alloys.

[60] Hobson ST, Braue EH, Lehnert EK, Klabunde KJ, Koper OP, Decker S (2002). Active topical skin protectants using reactive nanoparticles.

[61] Hobson ST, Braue EH, Shea K (2002). Active topical skin protectants using organic inorganic polysilsesquioxane materials.

[62] Hobson ST, Braue EH, Lehnert EK (2002). Active topical skin protectants using combinations of reactive nanoparticles and polyoxometalates or metal salts.

[63] Hurst CG, Sidell FR, Takafuji ET, Franz DR (1997). Decontamination. Warfare, Weaponry and the Casualty—Medical Aspects of Chemical and Biological Warfare.

[64] Food and Drug Administration (2003). Skin decontamination lotion cleared for military use. FDA Consumer.

[65] Van Hooidonk C, Ceulen BI, Bock J, van Genderen J (1983). CW agents and the skin. Penetration and decontamination.

[66] Smith HW, Clowes GHA, Marshall EK (1991). On dichloroethylsulphide (mustard gas), IV: the mechanism of absorption by the skin. J Pharm Exp Ther.

[67] Cullumbine H (1942). Prevention of Vesication.

[68] Cullumbine H, Liddell HF (1946). The penetration of antigas ointments into skin. Br J Dermatol.

[69] Smith WJ, Babin MC, Kiser RC, Casillas RP, Salem H, Katz SA (2003). Development of medical countermeasures to sulfur mustard vesication. Alternative Toxicological Methods.

[70] Babin MC, Ricketts K, Skvorak JP, Gazaway M, Mitcheltree LW, Casillas RP (2000). Systemic administration of candidate antivesicants to protect against topically applied sulfur mustard in the mouse ear vesicant model (MEVM). J Appl Toxicol.

[71] Casillas RP, Kiser RC, Truxall JA (2000). Therapeutic approaches to dermatotoxicity by sulfur mustard, I: modulation of sulfur mustard-induced cutaneous injury in the mouse ear vesicant model. J Appl Toxicol.

[72] Ruhl CM, Park SJ, Danisa O (1994). A serious skin sulfur mustard burn from an artillery shell. J Emerg Med.

[73] Kadivar H, Adams SC (1991). Treatment of chemical and biological warfare injuries: insights derived from the 1984 Iraqi attack on Majnoon Island. Mil Med.

[74] US Army Medical Research Institute of Chemical Defense (2000). Field Management of Chemical Casualties Handbook.

[75] US Army Medical Research Institute of Chemical Defense (2000). Medical Management of Chemical Casualties Handbook.

[76] Vesicants (blister agents) (1996). NATO Handbook on the Medical Aspects of NBC Defensive Operations, III: Chemical [AMedP-6(B) part III]. https://www.mega.nu/nbcmans.html.

[77] Marsden AK, Settle JAD (1996). Accident department. Principles and Practice of Burns Management.

[78] Johnson RM, Richard R (2003). Partial-thickness burns: identification and management. Adv Skin Wound Care.

[79] Arturson G, Settle JAD (1996). Local effects. Principles and Practice of Burns Management.

[80] Settle JAD, Settle JAD (1996). Principals of replacement fluid therapy. Principles and Practice of Burns Management.

[81] Brisebois RJ (2003). Fluid resuscitation in the Canadian forces. J Trauma.

[82] Thomas SJ, Kramer GC, Herndon DN (2003). Burns: military options and tactical solutions. J Trauma.

[83] Graham JS, Schomacker KT, Glatter RD, Briscoe CM, Braue EH, Squibb KS (2002). Bioengineering methods employed in the study of wound healing of sulfur mustard burns. Skin Res Tech.

[84] Belcher HJCR, Settle JAD (1996). Immunological responses. Principles and Practice of Burns Management.

[85] Laitung G, Settle JAD (1996). Metabolic responses and requirements. Principles and Practice of Burns Management.

[86] Burnside I, Settle JAD (1996). Psychological aspects of burn injuries. Principles and Practice of Burns Management.

[87] Hebda PA, Klingbeil CK, Abraham JA, Fiddes JC (1990). Basic fibroblast growth factor stimulation of epidermal wound healing in pigs. J Invest Dermatol.

[88] Montagna W, Yun JS (1964). The skin of the domestic pig. J Invest Dermatol.

[89] Swindle MM, Tumbleson ME (1986). Porcine models in surgical research: an overview. Swine in Biomedical Research.

[90] Dick IP, Scott RC (1992). Pig ear skin as an in-vitro model for human skin permeability. J Pharm Pharmacol.

[91] Paddock HN, Schultz GS, Mast BA, DiPietro LA, Burns AL (2003). Methods in reepithelialization. Wound Healing.

[92] Singer AJ, McClain SA, DiPietro LA, Burns AL (2003). A porcine burn model. Wound Healing.

[93] US Environmental Protection Agency (1992). Interim Guidance for Dermal Exposure Assessment.

[94] Klain GJ, Bonner SJ, Bell WG, Tumbleson ME (1986). The distribution of selected metabolic processes in the pig and human skin. Swine in Biomedical Research.

[95] Meyer W, Schwarz R, Neurand K (1978). Current Problems in Dermatology.

[96] Chilcott RP, Jenner J, Hotchkiss SA, Rice P (2001). In vitro skin absorption and decontamination of sulphur mustard: comparison of human and pig-ear skin. J Appl Toxicol.

[97] Lindsay CD, Rice P (1995). Changes in connective tissue macromolecular components of Yucatan mini-pig skin following application of sulphur mustard vapor. Hum Exp Toxicol.

[98] Smith KJ, Graham JS, Hamilton TA, Skelton HG, Petrali JP, Hurst CG (1997). Immunohistochemical studies of basement membrane proteins and proliferation and apoptosis markers in sulfur mustard induced cutaneous lesions in weanling pigs. J Dermatol Sci.

[99] Rice P The use of dermabrasion to accelerate the naturally slow rate of epidermal healing mustard injuries in pigs.

[100] Graham JS, Smith KJ, Braue EH (1997). Improved healing of sulfur mustard-induced cutaneous lesions in the weanling pig by pulsed CO_2_ laser debridement. J Toxicol Cutan Ocul Toxicol.

[101] Brown RFR, Rice P, Bennett NJ (1998). The use of laser Doppler imaging as an aid in clinical management decision making in the treatment of vesicant burns. Burns.

[102] Chilcott RP, Brown RFR, Rice P (2000). Non-invasive quantification of skin injury resulting from exposure to sulphur mustard and Lewisite vapours. Burns.

[103] Graham JS, Reid FM, Smith JR (2000). A cutaneous full-thickness liquid sulfur mustard burn model in weanling swine: clinical pathology and urinary excretion of thiodiglycol. J Appl Toxicol.

[104] Logan TP, Graham JS, Martin JL, Zallnick JE, Jakubowski EM, Braue EH (2000). Detection and measurement of sulfur mustard (HD) offgassing from the weanling pig following exposure to saturated sulfur mustard vapor. J Appl Toxicol.

[105] Reid FM, Graham JS, Niemuth NA, Singer AW, Janny SJ (2000). Sulfur mustard-induced skin burns in weanling swine evaluated clinically and histopathologically. J Appl Toxicol.

[106] Rice P, Brown RFR, Lam DGK, Chilcott RP, Bennett NJ (2000). Dermabrasion—a novel concept in the surgical management of sulphur mustard injuries. Burns.

[107] Rice P, Bennett NJ, Lam DGK, Brown RFR (2000). The role of dermabrasion in Lewisite-induced skin injury.

[108] Miller TL, Graham JS, Hayes TL, Reid FM (2001). Stability of sulfur mustard in vehicles suitable for cutaneous exposure of swine. J Toxicol Cutan Ocul Toxicol.

[109] Lam DGK, Rice P, Brown RFR (2002). The treatment of Lewisite burns with laser debridement—“lasablation.”. Burns.

[110] Chilcott RP, Stubbs B, Ashley Z (2001). Habituating pigs for in-pen, non-invasive biophysical skin analysis. Lab Anim.

[111] US Food and Drug Administration Draft guidance for industry: chronic cutaneous ulcer and burn wounds—developing products for treatment. http://www.fda.gov/cder/guidance/3226dft.pdf.

[112] Arroyo CM, Schafer RJ, Kurt EM, Broomfield CA, Carmichael AJ (2000). Response of normal human keratinocytes to sulfur mustard: cytokine release. J Appl Toxicol.

[113] Ricketts KM, Santai CT, France JA (2000). Inflammatory cytokine response in sulfur mustard-exposed mouse skin. J Appl Toxicol.

[114] Amir A, Chapman S, Kadar T, Gozes Y, Sahar R, Allon N (2000). Sulfur mustard toxicity in macrophages: effect of dexamethasone. J Appl Toxicol.

[115] Amir A, Turetz J, Chapman S (2000). Beneficial effects of topical anti-inflammatory drugs against sulfur-mustard-induced ocular lesions in rabbits. J Appl Toxicol.

[116] Husain K, Dube SN, Sugendran K, Singh R, Das Gupta S, Somani HD (1996). Effect of topically applied sulphur mustard on antioxidant enzymes in blood cells and body tissues of rats. J Appl Toxicol.

[117] Kumar O, Sugendran K, Vijayaraghavan R (2001). Protective effect of various antioxidants on the toxicity of sulphur mustard administered to mice by inhalation or percutaneous routes. Chem Biol Interact.

[118] Naghii MR (2002). Sulfur mustard intoxication, oxidative stress and antioxidants. Mil Med.

[119] Helfman T, Ovington L, Falanga V (1994). Occlusive dressings and wound healing. Clin Dermatol.

[120] Chang H, Wind S, Kerstein MD (1996). Moist wound healing. Dermatol Nurs.

[121] Kerstein MD (1995). Moist wound healing: the clinical perspective. Ostomy Wound Manage.

[122] Field CK, Kerstein MD (1994). Overview of wound healing in a moist environment. Am J Surg.

[123] Koga M (1988). Epidermal grafting using the tops of suction blisters in the treatment of vitiligo. Arch Dermatol.

[124] Tang WYM, Chan LY, Lo KK (1998). Treatment of vitiligo with autologous epidermal transplantation using the roofs of suction blisters. Hong Kong Med J.

[125] Kiistala U, Mustakallio KK (1964). In-vivo separation of epidermis by production of suction blisters. Lancet.

[126] Arturson G, Settle JAD (1996). Mechanism of injury. Principles and Practice of Burns Management.

[127] Bircher AJ, Serup J, Jemec GBE (1995). Laser Doppler measurement of skin blood flux: variation and validation. Handbook of Non-invasive Methods and the Skin.

[128] Belcaro G, Nicolaides AN, Serup J, Jemec GBE (1995). Laser-Doppler flowmetry: principles of technology and clinical applications. Handbook of Non-invasive Methods and the Skin.

[129] Wardell K, Andersson T, Anderson C (1996). Analysis of laser Doppler perfusion images of experimental irritant skin reactions. Skin Res Technol.

[130] Linden M, Wardell K, Andersson T, Anderson C (1997). High resolution laser Doppler perfusion imaging for the investigation of blood circulatory changes after microdialysis probe insertion. Skin Res Technol.

[131] Krogstad A-L, Pegenius G, Elam M (1996). Visual scoring and laser Doppler perfusion imaging of skin irritancy induced by different nicotine patches. Skin Res Technol.

[132] Wardell K, Nilsson G, Serup J, Jemec GBE (1995). Laser Doppler imaging of skin. Handbook of Non-invasive Methods and the Skin.

[133] Alsbjorn B, Micheels J, Sorensen B (1984). Laser Doppler flowmetry measurements of superficial dermal, deep dermal and subdermal burns. Scand J Plast Reconstr Surg.

[134] Blomgren I, Bagge U (1984). Postburn blood flow, edema and survival of the hairy mouse ear after injury at different temperatures. Scand J Plast Reconstr Surg.

[135] Garner WL, Thomson PD, Moore NP, Rodriguez JL, Smith DJ (1993). Effect of triglycyl-lysine-vasopressin on skin blood flow and blood loss during wound excision in patients with burns. J Burn Care Rehabil.

[136] Micheels J, Alsbjorn B, Sorensen B (1984). Clinical use of laser Doppler flowmetry in a burns unit. Scand J Plast Reconstr Surg.

[137] Niazi ZBM, Essex TJH, Papini R, Scott D, McLean NR, Black MJM (1993). New laser Doppler scanner, a valuable adjunct in burn depth assessment. Burns.

[138] O'Reilly TJ, Spence RJ, Taylor RM, Scheulen JJ (1989). Laser Doppler flowmetry evaluation of burn wound depth. J Burn Care Rehabil.

[139] Regas FC, Ehrlich HP (1992). Elucidating the vascular response to burns with a new rat model. J Trauma.

[140] Shakespeare PG (1992). Looking at burn wounds: the A.B. Wallace memorial lecture 1991. Burns.

[141] Waxman K (1989). Heated laser Doppler flow measurements to determine depth of burn injury. Am Surg.

[142] Heimbach D, Engrav L, Grube B, Marvin J (1992). Burn depth: a review. World J Surg.

[143] Green HA, Bua D, Anderson RR, Nishioka NS (1992). Burn depth estimation using indocyanine green fluorescence. Arch Dermatol.

[144] Black KS, Hewitt CW, Miller DM (1986). Burn depth evaluation with fluorometry: is it really definitive?. J Burn Care Rehab.

[145] Schomacker KT, Torri A, Sandison DR, Sheridan RL, Nishioka NS (1997). Biodistribution of indocyanine green in a porcine burn model: light and fluorescence microscopy. J Trauma.

[146] Jerath MR, Schomacker KT, Sheridan RL, Nishioka NS (1999). Burn wound assessment in porcine skin using indocyanine green fluorescence. J Trauma.

[147] Holm C, Mayr M, Tegeler J, Becker A, Pfeiffer U, Muhlbauer W (2003). Laser–induced fluorescence of indocyanine green: plastic surgical applications. Eur J Plast Surg.

[148] Holm C, Mayr M, Hofter E, Becker A, Pfeiffer UJ, Muhlbauer W (2002). Intraoperative evaluation of skin-flap viability using laser-induced fluorescence of indocyanine green. Br J Plast Surg.

[149] Sheridan RL, Schomacker KT, Lucchina LC (1995). Burn depth estimation by use of indocyanine green fluorescence: initial human trial. J Burn Care Rehabil.

[150] Chilcott RP, Dalton CH, Emmanuel AJ, Allen CE, Bradley ST (2002). Transepidermal water loss does not correlate with skin barrier function in vitro. J Invest Dermatol.

[151] Kjellstrom BT, Persson JKE, Runn P (1997). Surgical treatment of skin lesions induced by sulfur mustard (“mustard gas”)—an experimental study in the guinea pig. Ann Acad Med Singapore.

[152] Eldad A, Weinberg A, Breiterman S, Chaouat M, Palanker D, Ben-Bassat H (1998). Early nonsurgical removal of chemically injured tissue enhances wound healing in partial-thickness burns. Burns.

[153] Fitzpatrick RE, Goldman MP, Satur NM, Tope WD (1996). Pulsed carbon dioxide laser resurfacing of photoaged facial skin. Arch Dermatol.

[154] Hruza GJ (1996). Laser skin resurfacing. Arch Dermatol.

[155] Schoenrock LD, Chernoff WG, Rubach BW (1995). Cutaneous UltraPulse® laser resurfacing of the eyelids. Int J Aesth Restor Surg.

[156] Stellar S, Levine N, Ger R, Levenson HD (1973). Laser excision of acute third degree burns followed by immediate autograft replacement: an experimental study in the pig. J Trauma.

[157] Fidler JP, Law E, Rockwell RJ, MacMillan BG (1974). Carbon dioxide laser excision of acute burns with immediate autografting. J Surg Res.

[158] Levine N, Ger R, Stellar S, Levenson HD (1974). Use of a carbon dioxide laser for the debridement of third degree burns. Ann Surg.

[159] Levine NS, Salisbury RE, Peterson HD, Pruitt BA (1975). Clinical evaluation of the carbon dioxide laser for burn wound excisions: a comparison of the laser, scalpel and electrocautery. J Trauma.

[160] Green HA, Burd EE, Nishioka NS, Compton CC (1993). Skin graft take and healing following 193-nm excimer, continuous-wave carbon dioxide (CO_2_), pulsed CO_2_, or pulsed holmium:YAG laser ablation of the graft bed. Arch Dermatol.

[161] Green HA, Burd E, Nishioka NS, Brüggemann U, Compton CC (1992). Middermal wound healing. Arch Dermatol.

[162] Walsh JT, Flotte TJ, Anderson RR, Deutsch TF (1988). Pulsed CO_2_ laser tissue ablation: effect of tissue type and pulse duration on thermal damage. Lasers Surg Med.

[163] Fitzpatrick RE, Goldman MP (1995). Advances in carbon dioxide laser surgery. Clin Dermatol.

[164] Fitzpatrick RE, Goldman MP, Ruiz-Esparza J (1994). Clinical advantage of the CO_2_ laser superpulsed mode. J Dermatol Surg Oncol.

[165] Hobbs ER, Bailin PL, Wheeland RG, Ratz JL (1987). Superpulsed lasers: minimizing thermal damage with short duration, high irradiance pulses. J Dermatol Surg Oncol.

[166] Green HA, Domankevitz Y, Nishioka NS (1990). Pulsed carbon dioxide laser ablation of burned skin: *in vitro* and *in vivo* analysis. Lasers Surg Med.

[167] Domankevitz Y, Nishioka NS (1997). Effects of a rapidly scanned carbon dioxide laser on porcine dermis. J Burn Care Rehabil.

[168] Schomacker KT, Walsh JT, Flotte TJ, Deutsch TF (1990). Thermal damage produced by high irradiance continuous wave CO_2_ laser cutting of tissue. Lasers Surg Med.

[169] Glatter RD, Goldberg JS, Schomacker KT (1998). Carbon dioxide laser ablation with immediate autografting in a full-thickness porcine burn model. Ann Surg.

[170] Sheridan RL, Lydon MM, Petras LM (1999). Laser ablation of burns: initial clinical trial. Surgery.

[171] Acland KM, Barlow RJ (2000). Lasers for the dermatologist. Br J Dermatol.

[172] Tanzi EL, Alster TS (2003). Side effects and complications of variable-pulsed erbium:yttrium-aliminum-garnet laser skin resurfacing: extended experience with 50 patients. Plast Reconstr Surg.

[173] Tanzi EL, Alster TS (2003). Single-pass carbon dioxide versus multiple-pass Er:YAG laser skin resurfacing: a comparison of postoperative wound healing and side-effect rates. Dermatol Surg.

[174] Jeong JT, Park JH, Kye YC (2003). Resurfacing of pitted facial acne scars using Er:YAG laser with ablation and coagulation mode. Aesthetic Plast Surg.

[175] Reynolds N, Cawrse N, Burge T, Kenealy J (2003). Debridement of a mixed partial and full-thickness burn with an erbium:YAG laser. Burns.

[176] Sawyer TW, Nelson P, Hill I, Conley JD, Blohm K, Davidson C (2002). Therapeutic effects of cooling swine skin exposed to sulfur mustard. Mil Med.

[177] Wolff H, Hansson C (2003). Larval therapy—an effective method of ulcer debridement. Clin Exp Dermatol.

[178] Wollina U, Karte K, Herold C, Looks A (2000). Biosurgery in wound healing—the renaissance of maggot therapy. J Eur Acad Dermatol Venereol.

[179] Ballard K, Baxter H (2000). Developments in wound care for difficult to manage wounds. Br J Nurs.

[180] Courtenay M (1999). The use of larval therapy in wound management in the UK. J Wound Care.

[181] Rayner K (1999). Larval therapy in wound debridement. Prof Nurse.

[182] Thomas S, Andrews A, Jones M (1998). The use of larval therapy in wound management. J Wound Care.

[183] Waters J (1998). The benefits of larval therapy in wound care. Nurs Times.

[184] Prete PE (1997). Growth effects of *Phaenicia sericata* larval extracts on fibroblasts: mechanism for wound healing by maggot therapy. Life Sci.

[185] Singhal A, Reis ED, Kerstein MD (2001). Options for nonsurgical debridement of necrotic wounds. Adv Skin Wound Care.

[186] Ozcan C, Ergun O, Celik A, Corduk N, Ozok G (2002). Enzymatic debridement of burn wound with collagenase in children with partial-thickness burns. Burns.

[187] Orgill DP, Liu PY, Ritterbush LS, Skrabut EM, Samuels JA, Shames SL (1996). Debridement of porcine burns with a highly purified, ananain-based cysteine protease preparation. J Burn Care Rehabil.

[188] Hansbrough JF, Achauer B, Dawson J (1995). Wound healing in partial-thickness burn wounds treated with collagenase ointment versus silver sulfadiazine cream. J Burn Care Rehabil.

[189] Soroff HS, Sasvary DH (1994). Collagenase ointment and polymyxin B sulfate/bacitracin spray versus silver sulfadiazine cream in partial-thickness burns: a pilot study. J Burn Care Rehabil.

[190] Ahle NW, Hamlet MP (1987). Enzymatic frostbite eschar debridement by bromelain. Ann Emerg Med.

[191] Houck JC, Chang CM, Klein G (1983). Isolation of an effective debriding agent from the stems of pineapple plants. Int J Tissue React.

[192] Levenson HD, Gruber DK, Gruber C, Lent R, Seifter E (1981). Chemical debridement of burns: mercaptans. J Trauma.

[193] Gant TD (1980). The early enzymatic debridement and grafting of deep dermal burns to the hand. Plast Reconstr Surg.

[194] Klasen HJ (2000). A review on the nonoperative removal of necrotic tissue from burn wounds. Burns.

[195] Braue EH, Nalls CR, Way RA, Zallnick JE, Reider RG, Mitcheltree LW (1997). Nikolsky's sign: a novel way to evaluate damage at the dermal-epidermal junction. Skin Res Technol.

[196] Goodman H (1953). Nikolsky sign. Arch Dermatol Syphilol.

[197] Brown RFR, Rice P (1997). Histopathological changes in Yukatan minipig skin following challenge with sulfur mustard. A sequential study of the first 24 hours following challenge. Int J Exp Pathol.

[198] Yin HQ, Langford R, Burrell RE (1999). Comparative evaluation of the antimicrobial activity of ACTICOAT antimicrobial barrier dressing. J Burn Care Rehabil.

[199] Thomas S, McCubbin P (2003). A comparison of the antimicrobial effects of four silver-containing dressings on three organisms. J Wound Care.

[200] O'Neill MA, Vine GJ, Beezer AE (2003). Antimicrobial properties of silver-containing wound dressings: a microcalorimetric study. Int J Pharm.

[201] Olson ME, Wright JB, Lam K, Burrell RE (2000). Healing of porcine donor sites covered with silver-coated dressings. Eur J Surg.

[202] Demling RH, DeSanti MDL (2002). The rate of re-epithelialization across meshed skin grafts is increased with exposure to silver. Burns.

[203] Sullivan TP, Eaglstein WH, Davis SC, Mertz BA (2001). The pig as a model for human wound healing. Wound Rep Reg.

[204] McGrath MH (1990). Peptide growth factors and wound healing. Clin Plast Surg.

[205] Bennett NT, Schultz GS (1993). Growth factors and wound healing: biochemical properties of growth factors and their receptors. Am J Surg.

[206] Bennett NT, Schultz GS (1993). Growth factors and wound healing, II: role in normal and chronic wound healing. Am J Surg.

[207] Dahn MS (1998). The role of growth factors in wound management of diabetic foot ulcers. Fed Pract.

[208] Greenhalgh DG (1996). The role of growth factors in wound healing. J Trauma.

[209] Nanney LB, King LE, Clark RAF (1996). Epidermal growth factor and transforming growth factor-alpha. The Molecular and Cellular Biology of Wound Repair.

[210] Brown GL, Nanney LB, Griffen J (1989). Enhancement of wound healing by topical treatment with epidermal growth factor. N Engl J Med.

[211] Nakamura M, Nishida T (2003). Potentiation by cyclic AMP of the stimulatory effect of epidermal growth factor on corneal epithelial migration. Cornea.

[212] Fujisawa K, Miyamoto Y, Nagayama M (2003). Basic fibroblast growth factor and epidermal growth factor reverse impaired ulcer healing of the rabbit oral mucosa. J Oral Pathol Med.

[213] Tsang MW, Wong WK, Hung CS (2003). Human epidermal growth factor enhances healing of diabetic foot ulcers. Diabetes Care.

[214] Gope R (2002). The effect of epidermal growth factor & platelet-derived growth factors on wound healing process. Indian J Med Res.

[215] Ishikawa T, Terai H, Yamamoto T, Harada K, Kitajima T (2003). Delivery of a growth factor fusion protein having collagen-binding activity to wound tissues. Artif Organs.

[216] Lee J (2002). Formulation development of epidermal growth factor. Pharmazie.

[217] Kim MJ, Jun RM, Kim WK (2001). Optimal concentration of human epidermal growth factor (hEGF) for epithelial healing in experimental corneal alkali wounds. Curr Eye Res.

[218] Cribbs RK, Luquette MH, Besner GE (1998). Acceleration of partial-thickness burn wound healing with topical application of heparin-binding EGF-like growth factor (HB-EGF). J Burn Care Rehabil.

[219] Danilenko DM, Ring BD, Tarpley JE (1995). Growth factors in porcine full and partial-thickness burn repair. Differing targets and effects of keratinocyte growth factor, platelet-derived growth factor-BB, epidermal growth factor, and neu differentiation factor. Am J Pathol.

[220] Gonul B, Erdogan D, Ozogul C, Koz M, Babul A, Celebi N (1995). Effect of EGF dosage forms on alkali burned corneal wound healing of mice. Burns.

[221] Cohen IK, Crossland MC, Garrett A, Diegelmann RF (1995). Topical application of epidermal growth factor onto partial-thickness wounds in human volunteers does not enhance reepithelialization. Plast Reconstr Surg.

[222] Wenczak BA, Lynch JB, Nanney LB (1992). Epidermal growth factor receptor distribution in burn wounds. Implications for growth factor-mediated repair. J Clin Invest.

[223] Brown GL, Curtsinger L, Jurkiewicz MJ, Nahai F, Schultz G (1991). Stimulation of healing chronic wounds by epidermal growth factor. Plast Reconstr Surg.

[224] Wang HJ, Wan HL, Yang DS, Chen TM, Chang DM (1996). Acceleration of skin graft healing by growth factors. Burns.

[225] Heldin C-H, Westermark B, Clark RAF (1996). Role of platelet-derived growth factor in vivo. The Molecular and Cellular Biology of Wound Repair.

[226] Saba AA, Freedman BM, Gaffield JW, Mackay DR, Ehrlich HP (2002). Topical platelet-derived growth factor enhances wound closure in the absence of wound contraction: an experimental and clinical study. Ann Plast Surg.

[227] Lariviere B, Rouleau M, Picard S, Beaulieu AD (2003). Human plasma fibronectin potentiates the mitogenic activity of platelet-derived growth factor and complements its wound healing effects. Wound Repair Regen.

[228] Bennett SP, Griffiths GD, Schor AM, Leese GP, Schor SL (2003). Growth factors in the treatment of diabetic foot ulcers. Br J Surg.

[229] Boykin JV (2000). The nitric oxide connection: hyperbaric oxygen therapy, becaplermin and diabetic ulcer management. Adv Skin Wound Care.

[230] Cui Y, Urschel JD, Petrelli NJ (1999). The effect of keratinocyte growth factor-2 on esophagogastric anastomotic wound healing in rats. Int J Surg Invest.

[231] Gamady A, Koren R, Ron D, Liberman UA, Ravid A (2003). Vitamin D enhances mitogenesis mediated by keratinocyte growth factor receptor in keratinocytes. J Cell Biochem.

[232] Galiacy S, Planus E, Lepetit H (2003). Keratinocyte growth factor promotes cell motility during alveolar epithelial repair in vitro. Exp Cell Res.

[233] Smith PD, Polo M, Soler PM (2000). Efficacy of growth factors in the accelerated closure of interstices in explanted meshed human skin grafts. J Burn Care Rehabil.

[234] Yoshida S, Yamaguchi Y, Itami S (2003). Neutralization of hepatocyte growth factor leads to retarded cutaneous wound healing associated with decreased neovascularization and granulation tissue formation. J Invest Dermatol.

[235] Koshizuka S, Kanazawa K, Kobayashi N (1997). The beneficial effects of human insulin-like growth factor-I (IGF-I) on wound healing in severely wounded senescent mice. Surg Today.

[236] Sorensen OE, Cowland JB, Theilgaard-Monch K, Liu L, Ganz T, Borregaard N (2003). Wound healing and expression of antimicrobial peptides/polypeptides in human keratinocytes, a consequence of common growth factors. J Immunol.

[237] Goren I, Pfeilschifter J, Frank S (2003). Determination of leptin signaling pathways in human and murine keratinocytes. Biochem Biophys Res Commun.

[238] Frank S, Stallmeyer B, Kampfer H, Kolb N, Pfeilschifter J (2000). Leptin enhances wound re-epithelialization and constitutes a direct function of leptin in skin repair. J Clin Invest.

[239] Roberts AB, Sporn MB, Clark RAF (1996). Transforming growth factor-beta. The Molecular and Cellular Biology of Wound Repair.

[240] Groves RW, Schmidt-Lucke JA (2000). Recombinant human GM-CSF in the treatment of poorly healing wounds. Adv Skin Wound Care.

[241] Abraham JA, Klagsbrun M, Clark RAF (1996). Modulation of wound repair by members of the fibroblast growth factor family. The Molecular and Cellular Biology of Wound Repair.

[242] Clark RAF, Clark RAF (1996). Wound repair. Overview and general considerations. The Molecular and Cellular Biology of Wound Repair.

[243] Yamada KM, Clark RAF, Clark RAF (1996). Provisional matrix. The Molecular and Cellular Biology of Wound Repair.

[244] Woodley DT, Clark RAF (1996). Reepithelialization. The Molecular and Cellular Biology of Wound Repair.

[245] Sheridan RL, Tompkins RG (1999). Skin substitutes in burns. Burns.

[246] Sheridan RL, Moreno C (2001). Skin substitutes in burns. Burns.

[247] Shakespeare P (2001). Skin substitutes—benefits and costs. Burns.

[248] Sheridan RL, Morgan JR, Cusick JL, Petras LM, Lydon MM, Tompkins RG (2001). Initial experience with a composite autologous skin substitute. Burns.

[249] Shakespeare P (2001). Burn wound healing and skin substitutes. Burns.

[250] Boyce ST (2001). Design principles for composition and performance of cultured skin substitutes. Burns.

[251] Balasubramani M, Kumar TR, Babu M (2001). Skin substitutes: a review. Burns.

[252] Kearney JN (2001). Clinical evaluation of skin substitutes. Burns.

[253] Hansen SL, Voigt DW, Wiebelhaus P, Paul CN (2001). Using skin replacement products to treat burns and wounds. Adv Wound Care.

[254] Martin LK, Kirsner RS (2002). Use of a meshed bilayered cellular matrix to treat a venous ulcer. Adv Skin Wound Care.

[255] Phillips TJ, Manzoor J, Rojas A (2002). The longevity of a bilayered skin substitute after application to venous ulcers. Arch Dermatol.

[256] Bello YM, Falabella AF (2002). The role of graftskin (Apligraf) in difficult-to-heal venous leg ulcers. J Wound Care.

[257] Paquette D, Falanga V (2002). Leg ulcers. Clin Geriatr Med.

[258] Long RE, Falabella AF, Valencia I, Eaglstein WH, Kirsner RS (2001). Treatment of refractory, atypical lower extremity ulcers with tissue-engineered skin (Apligraf). Arch Dermatol.

[259] Brem H, Balledux J, Sukkarieh T, Carson P, Falanga V (2001). Healing of venous ulcers of long duration with a bilayered living skin substitute: results from a general surgery and dermatology department. Dermatol Surg.

[260] Phillips TJ (2001). Current approaches to venous ulcers and compression. Dermatol Surg.

[261] Veves A, Falanga V, Armstrong DG, Sabolinski ML (2001). Graftskin, a human skin equivalent, is effective in the management of noninfected neuropathic diabetic foot ulcers: a prospective randomized multicenter clinical trial. Diabetes Care.

[262] Edmonds M, Bates M, Doxford M, Gough A, Foster A (2000). New treatments in ulcer healing and wound infection. Diabetes Metab Res Rev.

[263] Schonfeld WH, Villa KF, Fastenau JM, Mazonson PD, Falanga V (2000). An economic assessment of Apligraf (Graftskin) for the treatment of hard-to-heal venous leg ulcers. Wound Repair Regen.

[264] Brem H, Balledux J, Bloom T, Kerstein MD, Hollier L (2000). Healing of diabetic foot ulcers and pressure ulcers with human skin equivalent: a new paradigm in wound healing. Arch Surg.

[265] Falanga V, Sabolinski M (1999). A bilayered skin construct (Apligraf) accelerates complete closure of hard-to-heal venous ulcers. Wound Repair Regen.

[266] Dolynchuk K, Hull P, Guenther L (1999). The role of Apligraf in the treatment of venous leg ulcers. Ostomy Wound Manage.

[267] Falanga V (1998). Apligraf treatment of venous ulcers and other chronic wounds. J Dermatol.

[268] Boyce ST, Glatter R, Kitzmiller WJ (1995). Case studies: treatment of chronic wounds with cultured skin substitutes. Ostomy Wound Manage.

[269] Kirsner RS (1998). The use of Apligraf in acute wounds. J Dermatol.

[270] Vloemans AFPM, Schreinemachers MCJM, Middelkoop E, Kreis RW (2002). The use of glycerol-preserved allografts in the Beverwijk Burn Centre: a retrospective study. Burns.

[271] Blome-Eberwein S, Jester A, Kuentscher M, Raff T, Germann G, Pelzer M (2002). Clinical practice of glycerol preserved allograft skin coverage. Burns.

[272] Moerman E, Middelkoop E, Mackie D, Groenevelt F (2002). The temporary use of allograft for complicated wounds in plastic surgery. Burns.

[273] Vloemans AFPM, Middelkoop E, Kreis RW (2002). A historical appraisal of the use of cryopreserved and glycerol-preserved allograft skin in the treatment of partial thickness burns. Burns.

[274] Dhennin C, Desbois I, Yassine AS, Benissad H, Lignee J (2002). Utilisation of glycerolised skin allografts in severe burns. Burns.

[275] Druecke D, Steinstraesser L, Homann HH, Steinau HU, Vogt PM (2002). Current indications for glycerol-preserved allografts in the treatment of burn injuries. Burns.

[276] Burd A (2002). Glycerolised allogenic skin: transplant or dressing? A medico-legal question. Burns.

[277] Mackie DP (2002). Postal survey on the use of glycerol-preserved allografts in clinical practice. Burns.

[278] Lawrence JC, Settle JAD (1996). Dressings for burns. Principles and Practice of Burns Management.

[279] Fenton RCO, Settle JAD (1996). Disfigurement and disablement. Principles and Practice of Burns Management.

[280] Broz L, Vogtova D, Konigova R (1999). Experience with banked skin in the Prague Burn Center. Acta Chir Plast.

[281] Phillips TJ, Kehinde O, Green H, Gilchrest BA (1989). Treatment of skin ulcers with cultured epidermal allografts. J Am Acad Dermatol.

[282] Simman R, TaliHDan R, Soroff HS, Hatch G, Simon M (1999). Cultured palmar keratinocytes after auto-engraftment to plantar surface maintain site and function specificity. Plast Reconstr Surg.

[283] Simman R, Priebe CJ, Simon M (2000). Reconstruction of aplasia cutis congenital of the trunk in a newborn infant using acellular allogenic dermal graft and cultured epithelial autografts. Ann Plast Surg.

[284] Hickerson WL, Compton C, Fletchall S, Smith LR (1994). Cultured epidermal allografts and allodermis combination for permanent burn wound coverage. Burns.

[285] Madden MR, Finkelstein JL, Staiano-Coico L (1986). Grafting of cultured allogeneic epidermis on second- and third-degree burn wounds on 26 patients. J Trauma.

[286] Kaawach WF, Oliver AM, Weiler-Mithoff E, Abramovich DR, Rayner CR (1991). Survival assessment of cultured epidermal allografts applied onto partial-thickness burn wounds. Br J Plast Surg.

[287] Teepe RG, Kreis RW, Koebrugge EJ (1990). The use of cultured autologous epidermis in the treatment of extensive burn wounds. J Trauma.

[288] Fabre JW (1991). Epidermal allografts. Immunol Lett.

[289] Phillips TJ, Provan A, Colbert D, Easley KW (1993). A randomized single-blind controlled study of cultured epidermal allografts in the treatment of split-thickness skin graft donor sites. Arch Dermatol.

[290] Soeda J, Inokuchi S, Ueno S (1993). Use of cultured human epidermal allografts for the treatment of extensive partial thickness scald burn in children. Tokai J Exp Clin Med.

[291] Fratianne R, Papay F, Housini I, Lang C, Schafer IA (1993). Keratinocyte allografts accelerate healing of split-thickness donor sites: applications for improved treatment of burns. J Burn Care Rehabil.

[292] Coleman JJ, Siwy BK (1992). Cultured epidermal autografts: a life-saving and skin-saving technique in children. J Pediatr Surg.

[293] Krupp S, Benathan M, Meuli M (1992). Current concepts in pediatric burn care: management of burn wounds with cultured epidermal autografts. Eur J Pediatr Surg.

[294] Frenk E, Benathan M (1992). Current concepts in pediatric burn care: morphology and biology of normal human skin and stratified cultures of epidermal cells for wound covering. Eur J Pediatr Surg.

[295] Haith LR, Patton ML, Goldman WT (1992). Cultured epidermal autograft and the treatment of the massive burn injury. J Burn Care Rehabil.

[296] Arons JA, Wainwright DJ, Jordon RE (1992). The surgical applications and implications of cultured human epidermis: a comprehensive review. Surgery.

[297] Munster AM (1992). Use of cultured epidermal autografts in ten patients. J Burn Care Rehabil.

[298] Nanchahal J, Dover R, Otto WR (2002). Allogeneic skin substitutes applied to burns patients. Burns.

[299] Yanaga H, Udoh Y, Yamauchi T (2001). Cryopreserved cultured epidermal allografts achieved early closure of wounds and reduced scar formation in deep partial-thickness burn wounds (DDB) and split-thickness skin donor sites of pediatric patients. Burns.

[300] Alvarez-Diaz C, Cuenca-Pardo J, Sosa-Serrano A, Juarez-Aguilar E, Marsch-Moreno M, Kuri-Harcuch W (2000). Controlled clinical study of deep partial-thickness burns treated with frozen cultured human allogeneic epidermal sheets. J Burn Care Rehabil.

[301] Loss M, Wedler V, Kunzi W, Meuli-Simmen C, Meyer VE (2000). Artificial skin, split-thickness autograft and cultured autologous keratinocytes combined to treat a severe burn injury of 95% of TBSA. Burns.

[302] Stoner ML, Wood FM (2000). The treatment of hypopigmented lesions with cultured epithelial autograft. J Burn Care Rehabil.

[303] Monstrey S, Beele H, Kettler M (1999). Allogeneic cultured keratinocytes vs cadaveric skin to cover wide-mesh autogenous split-thickness skin grafts. Ann Plast Surg.

[304] Raghunath M, Meuli M (1997). Cultured epithelial autografts: diving from surgery into matrix biology. Pediatr Surg Int.

[305] Nunez-Gutierrez H, Castro-Munozledo F, Kuri-Harcuch W (1996). Combined use of allografts and autograft epidermal cultures in therapy of burns. Plast Reconstr Surg.

[306] Wood F, Liddiard K, Skinner A, Ballentyne J (1996). Scar management of cultured epithelial autograft. Burns.

[307] Rivas-Torres MT, Amato D, Arambula-Alvarez H, Kuri-Harcuch W (1996). Controlled clinical study of skin donor sites and deep partial-thickness burns treated with cultured epidermal allografts. Plast Reconstr Surg.

[308] Putland M, Snelling CF, Macdonald I, Tron VA (1995). Histologic comparison of cultured epithelial autograft and meshed expanded split-thickness skin graft. J Burn Care Rehabil.

[309] Lopez Gutierrez JC, Ros Z, Vallejo D, Perdiguero M, Soto C, Tovar J (1995). Cultured epidermal autograft in the management of critical pediatric burn patients. Eur J Pediatr Surg.

[310] Brychta P, Suchanek I, Rihova H, Adler J, Komarkova J (1995). Cultured epidermal allografts for the treatment of deep dermal burns. Acta Chir Plast.

[311] Still JM, Orlet HK, Law EJ (1994). Use of cultured epidermal autografts in the treatment of large burns. Burns.

[312] Krupp S, Wiesner L, Krstic R, Pescia G, Winistorfer B (1994). Mid-term results with cultured epidermal autografts, allogenic skin transplants and cyclosporin A medication. Burns.

[313] Rheinwald JG, Green H (1975). Serial cultivation of strains of human epidermal keratinocytes: the formation of keratinizing colonies from single cells. Cell.

[314] Green H, Kehinde O, Thomas J (1979). Growth of cultured human epidermal cells into multiple epithelia suitable for grafting. Proc Natl Acad Sci.

[315] Braye F, Oddou L, Bertin-Maghit M (2000). Widely meshed autograft associated with cultured autologous epithelium for the treatment of major burns in children: report of 12 cases. Eur J Pediatr Surg.

[316] Svensjo T, Yao F, Pomahac B, Eriksson E (2001). Autologous keratinocyte suspensions accelerate epidermal wound healing in pigs. J Surg Res.

[317] Navarro FA, Stoner ML, Park CS (2000). Sprayed keratinocyte suspensions accelerate epidermal coverage in a porcine microwound model. J Burn Care Rehabil.

[318] Voigt M, Schauer M, Schaefer DJ, Andree C, Horch R, Stark GB (1999). Cultured epidermal keratinocytes on a microspherical transport system are feasible to reconstitute the epidermis in full-thickness wounds. Tissue Eng.

[319] Horch RE, Bannasch H, Kopp J, Andree C, Stark GB (1998). Single-cell suspensions of cultured human keratinocytes in fibrin-glue reconstitute the epidermis. Cell Transplant.

[320] Currie LJ, Martin R, Sharpe JR, James SE (2003). A comparison of keratinocyte cell sprays with and without fibrin glue. Burns.

[321] Wood F (2003). Clinical potential of autologous epithelial suspension. Wounds.

[322] Boyce ST, Supp AP, Harringer MD, Greenhalgh DG, Warden GD (1995). Topical nutrients promote engraftment and inhibit wound contraction of cultured skin substitutes in athymic mice. J Invest Dermatol.

[323] Zamboni WA, Browder LK, Martinez J (2003). Hyperbaric oxygen and wound healing. Clin Plast Surg.

[324] Kalliainen LK, Gordillo GM, Schlanger R, Sen CK (2003). Topical oxygen as an adjunct to wound healing: a clinical case series. Pathophysiology.

[325] Banwell PE, Teot L (2003). Topical negative pressure (TNP): the evolution of a novel wound therapy. J Wound Care.

[326] Mendez-Eastman S (1998). Negative pressure wound therapy. Plast Surg Nurs.

[327] Morykwas MJ, Argenta LC, Shelton-Brown EI, McGuirt W (1997). Vacuum-assisted closure: a new method for wound control and treatment. Animal studies and basic foundation. Ann Plast Surg.

[328] Joseph E, Hamori CA, Bergman S, Roaf E, Swann N, Anastasi GW (2000). A prospective randomized trial of vacuum-assisted closure versus standard therapy of chronic nonhealing wounds. Wounds.

[329] Morykwas MJ, Argenta LC (1997). Nonsurgical modalities to enhance healing and care of soft tissue wounds. J South Orthop Assoc.

[330] Mooney JF, Argenta LC, Marks MW, Morykwas MJ, DeFranzo AJ (2000). Treatment of soft tissue defects in pediatric patients using the V.A.C. system. Clin Orthop.

[331] Genecov DG, Schneider AM, Morykwas MJ, Parker D, White WL, Argenta LC (1998). A controlled subatmospheric pressure dressing increases the rate of skin graft donor site reepithelialization. Ann Plast Surg.

[332] Scherer LA, Shiver S, Chang M, Meredith JW, Owings JT (2002). The vacuum assisted closure device: a method of securing skin grafts and improving graft survival. Arch Surg.

[333] Morykwas MJ, David LR, Schneider AM (1999). Use of subatmospheric pressure to prevent progression of partial-thickness burns in a swine model. J Burn Care Rehabil.

